# Quantifying and predicting population connectivity of an outbreaking forest insect pest

**DOI:** 10.1007/s10980-021-01382-9

**Published:** 2021-12-23

**Authors:** Jeremy Larroque, Julian Wittische, Patrick M. A. James

**Affiliations:** 1 Department of Wildlife Sciences, University of Göttingen, Buesgenweg 3, 37077 Göttingen, Germany; 2 Department of Biological Sciences, Pavillon Marie-Victorin, Université de Montréal, 90 Vincent-d’Indy Avenue, Montreal, QC H2V 2S9 Canada; 3 Institute of Forestry and Conservation, John H. Daniels Faculty of Architecture, Landscape, and Design, University of Toronto, 33 Willcocks Street, Toronto, ON M5S 3E8 Canada

**Keywords:** Gene flow, Optimization, Spruce budworm, Global change, Resistance, Landscape genetics

## Abstract

**Context:**

Dispersal has a key role in the population dynamics of outbreaking species such as the spruce budworm (*Choristoneura fumiferana*) as it can synchronize the demography of distant populations and favor the transition from endemic to epidemic states. However, we know very little about how landscape structure influences dispersal in such systems while such knowledge is essential for better forecasting of spatially synchronous population dynamics and to guide management strategies.

**Objectives:**

We aimed to characterize the spatial environmental determinants of spruce budworm dispersal to determine how these features affect outbreak spread in Quebec (Canada). We then apply our findings to predict expected future landscape connectivity and explore its potential consequences on future outbreaks.

**Methods:**

We used a machine-learning landscape genetics approach on 447 larvae covering most of the outbreak area and genotyped at 3562 SNP loci to identify the main variables affecting connectivity.

**Results:**

We found that the connectivity between outbreak populations was driven by the combination of precipitation and host cover. Our forecasting suggests that between the current and next outbreaks, connectivity may increase between Ontario and Quebec, and might decrease in the eastern part, which could have the effect of limiting outbreak spread from Ontario and Quebec to the eastern provinces.

**Conclusions:**

Although we did not identify any discrete barriers, low connectivity areas might constrain dispersal in the current and future outbreaks and should in turn, be intensively monitored. However, continued sampling as the outbreak progresses is needed to confirm the temporal stability of the observed patterns.

**Supplementary Information:**

The online version contains supplementary material available at 10.1007/s10980-021-01382-9.

## Introduction

Insect outbreaks are a common disturbance in forest ecosystems and occur both periodically and synchronously over large geographic areas (Myers 1993; Peltonen et al. 2002). Spatially synchronous outbreak patterns have been documented for a variety of defoliating insects in North America, including the forest tent caterpillar (*Malacosoma disstria*, Hübner), the mountain pine beetle (*Dendroctonus ponderosae*, Hopkins), and several species of budworm such as the eastern spruce budworm (*Choristoneura fumiferana*, Clemens) and the jack-pine budworm (*Choristoneura pinus*, Freeman). During outbreaks, these insects can affect millions of hectares of susceptible forest (Cooke et al. 2007). In addition to their impact on ecosystem processes and services (e.g., risk of fire ignition, James et al. 2017; carbon sequestration, Kurz et al. 2008), these outbreaks can also have devastating socio-economic consequences (Gatto et al. 2009; Chang et al. 2012). As a landscape-scale contagious disturbance process (Peterson 2002), outbreak dynamics are strongly shaped by the spatial distribution and connectivity of susceptible hosts (Robert et al. 2020). However, we seldom know precisely how environmental heterogeneity and landscape connectivity influence the spatial dynamics of insect disturbances (e.g., Wittische et al. 2019).

The eastern spruce budworm (hereafter SBW) is a univoltine native lepidopteran that periodically outbreaks (every ~ 35 years) and defoliates large areas (> 10^6^ ha) of balsam fir (*Abies balsamea* (L.) Mill) and spruce (*Picea* spp.) forests in North America (Royama 1984). The severe economic consequences for forest industries and forestry‐dependent communities (Chang et al. 2012) make it the most significant forest insect disturbance in North America’s forests (Hardy et al. 1983).

In 2006, a new outbreak began on the north shore of the Saint Lawrence River in Quebec. Since then, the area affected has increased to > 13.5 million ha in Quebec (Ministère des Forêts de la Faune et des Parcs [MFFP] 2020) and is currently affecting other jurisdictions to the south (New Brunswick, Maine) and west (Ontario) with no signs of abating (Carleton et al. 2020).

Knowledge of SBW movement and especially dispersal capacity is essential for understanding SBW population ecology, predicting outbreak spread, and improving management strategies (Royama et al. 2005; Régnière and Nealis 2019). Dispersal can synchronize the demography of distant populations during outbreaks (Anderson and Sturtevant 2011; Larroque et al. 2019) and has the capacity to trigger the transition of spruce budworm populations from endemic to epidemic states (Régnière and Nealis 2019; Larroque et al. 2020). Consequently, it may be possible to slow outbreak spread and to mitigate the damage caused by outbreaks by limiting dispersal from attacked to unattacked areas, provided that potential source patches can be detected early and suitably treated (e.g., using *Btk*, Fuentealba et al. 2019). However, current knowledge regarding SBW dispersal is considered fragmented and insufficient to guide effective management interventions (Pureswaran et al. 2016; Johns et al. 2019).

Direct measurement of dispersal is costly and typically challenging to conduct (Ims and Andreassen 2005), especially for small flying insects such as the spruce budworm (Osborne et al. 2002). Even if SBW dispersal can be observed using weather surveillance radar (e.g., Boulanger et al. 2017), its effectiveness remains unknown as the source and destination of the dispersing individuals, their survival and reproductive status is uncertain (Régnière and Nealis 2019). Alternatively, spatial population genetic structure can be used to infer the frequency and routes of effective dispersal (Baguette et al. 2013). Significant genetic differentiation between populations generally indicates low levels of gene flow and limited dispersal. In contrast, the absence of genetic differentiation indicates high levels of gene flow and a high degree of effective dispersal between populations (Slatkin 1987; Rousset 1997). In a recent study of the ongoing outbreak, Larroque et al. (2019) identified high levels of SBW population genetic connectivity in Quebec. Populations associated with different outbreak patches all belonged to the same genetic cluster, even though these patches are separated by distances up to one thousand kilometers.

Further, finer scale examination of populations at the leading edge of the outbreak and into areas where the outbreak had not yet become established identified outbreak patches in Quebec and New Brunswick that were genetically differentiated despite being geographically close (*i.e.*, < 130 km) (Larroque et al. 2020). This contrasting result suggests that environmental heterogeneity might influence effective dispersal at finer spatial scales in these areas. Effective dispersal is the result of a complex interaction between an organism’s dispersal capacity and landscape connectivity. Here, landscape connectivity refers to the degree to which environmental conditions (e.g., land cover, topography, precipitation) impede or facilitate movement (Taylor et al. 1993). Landscape connectivity not only depends on characteristics of the environment, but also on the mobility of the organism, and the physiological cost and the reduction in survival associated with moving through a particular environment (Zeller et al. 2012). Landscape genetics, which combines population genetics, landscape ecology, and spatial statistics (Manel et al. 2003), can be used to infer landscape connectivity and identify which landscape features hinder or facilitate gene flow between populations (Balkenhol et al. 2015). This approach aims to characterize landscape resistance (the opposite of connectivity), that is, the cost to an organism to cross a landscape. Low resistance indicates ease of movement whereas high resistance indicates restricted movement (Zeller et al. 2012).

**Fig. 1:**
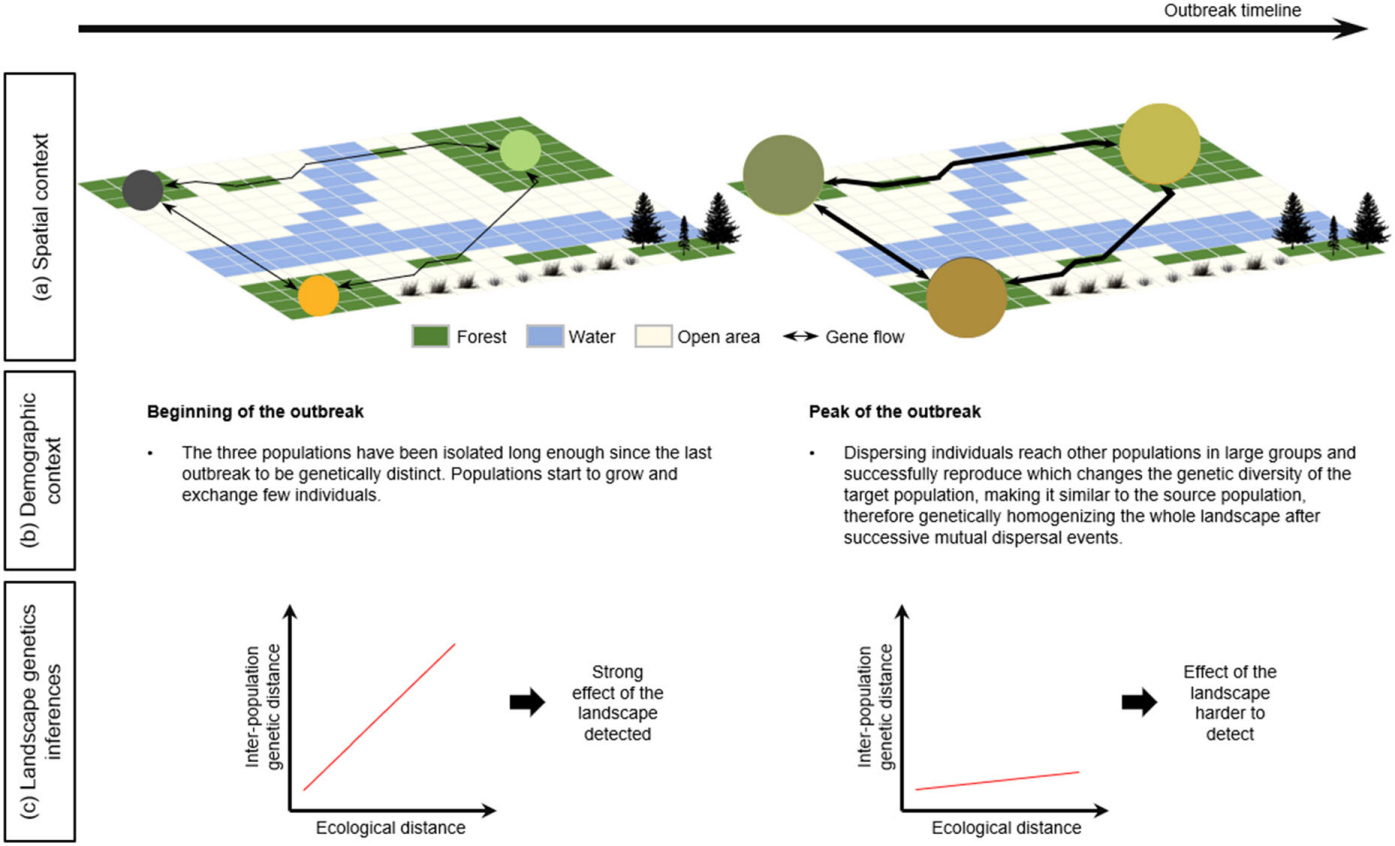
Illustration of the effect of the demographic context on landscape genetics inferences. Panel (**a**) illustrates three spatially distinct populations, and the landscape and the gene flow (*i.e.*, the genetic connectivity) among the populations at the beginning and at the peak of the outbreak. Panel (**b**) summarizes an example of the demographic context at the beginning and at the peak of the outbreak. Despite no change in the landscape effect, the number of successful dispersing individuals increases because population densities increase. Panel (**c**) illustrates the landscape genetics inferences at the beginning and at the peak of the outbreak. Greater gene flow resulted in reduced inter-population genetic distances that may mask the role of landscape resistance

Population genetic connectivity in outbreaking populations can be sensitive to demographic context (*i.e.*, outbreak stage; James et al. 2015; Larroque et al. 2019; Larroque et al. 2020). Consequently, landscape genetic inference in outbreaking systems can also be confounded due to the effects of population density and the shifting spatial extent of outbreaking populations (Fig. 1). These outbreak-related shifts may mask or dampen the influence of landscape heterogeneity on movement (Spear et al. 2010). During outbreak periods, increased population densities and density-dependent dispersal (Régnière and Nealis 2019) are expected to result in greater gene flow and reduced inter-population genetic distances relative to non-outbreak periods, even when considering constant landscape resistance. As a result, while the environmental features remain constant in time, the relationships between the inter-population genetic distances and the resistance-based effective distances might gradually decrease leading to the conclusion that the landscape has no effect on the effective dispersal (Fig. 1). Consideration of the demographic context is thus particularly important for cyclic irruptive species like the spruce budworm when evaluating the role of spatial heterogeneity in population connectivity.

Several environmental factors are known to affect SBW population dynamics and flight characteristics (Table 1). We hypothesize that these factors may also play a role in landscape genetic connectivity. Host‐species availability positively impacts SBW survival (Nealis and Régnière 2004b) and can influence the landing decision of dispersing SBW (Greenbank et al. 1980), while defoliation of hosts can promote flight behavior and emigration (Nealis and Régnière 2004a; Van Hezewijk et al. 2018). Temperature affects SBW phenology and survival (Régnière and You 1991); warm temperatures are known to increase flight activity (Sanders et al. 1978) while cool temperatures can trigger the moth landing (Sturtevant et al. 2013). Wind influences dispersal direction and distance (Greenbank et al. 1980; Anderson and Sturtevant 2011), and elevation can negatively affect outbreak expansion (Bouchard and Auger 2014). Finally, precipitation can limit moth flight (Greenbank et al. 1980) and force the descent of flying moths (Sturtevant et al. 2013). The impact of these factors on SBW effective dispersal has not been assessed, and the degree of functional connectivity between the established outbreak patches remains unknown. Identifying the most important drivers of SBW connectivity and thus, high connectivity areas, can help us to better understand the complex spatial and temporal dynamics of outbreaking forest insect pests and could also help guide management efforts.

**Table 1:** Description of landscape variables used for building landscape resistance surfaces and the associated hypotheses

Environmental variable	Description	Potential effect	Source
Host cover	Percentage of balsam fir + white spruce cover (%) per pixel	(+) Preference for high-cover stands can increase effective dispersal	NRCAN/CFS
Defoliation	Severity of the SBW defoliation (3 levels)	(+) Food-depleted areas can promote dispersal (−) Food-depleted areas can decrease the larvae survival	MFFP
Wind speed	Mean annual wind speed at 200 m a.s.l	(+) High wind speed can promote dispersal	Global Wind Atlas
Mean precipitation	Mean daily July precipitation (mm)	(+) High level of precipitation can increase larvae survival (−) High level of precipitation can decrease effective dispersal	NRCAN/CFS
Mean temperature	Mean daily July temperature (°C)	(+) Warm temperatures promote dispersal and increase larvae survival	NRCAN/CFS
Elevation	Digital elevation model (m)	(−) Relief could act as a barrier for wind-dispersed SBW	R package *elevatr*

In this paper, we investigate how landscape heterogeneity affects spruce budworm population genetic connectivity to better understand outbreak spread during the early stage of the outbreak, *i.e.*, before resistance patterns can be confounded by demographic processes. We used a machine-learning landscape genetics approach (Peterman 2018) that employs a genetic algorithm (Holland 1975) to first optimize the resistance of land cover and bioclimatic variables (Table 1) and then use these resistance surfaces to model landscape genetic connectivity. Additionally, as climate change is predicted to have large effects on forest productivity and natural ecosystems, in part as a result of changing dynamics between plants and their pests (Logan et al. 2003; Pureswaran et al. 2018b), we predicted future landscape connectivity in Quebec as it may manifest for future outbreaks.

## Materials and methods

### Study area and genetic data

**Fig. 2:**
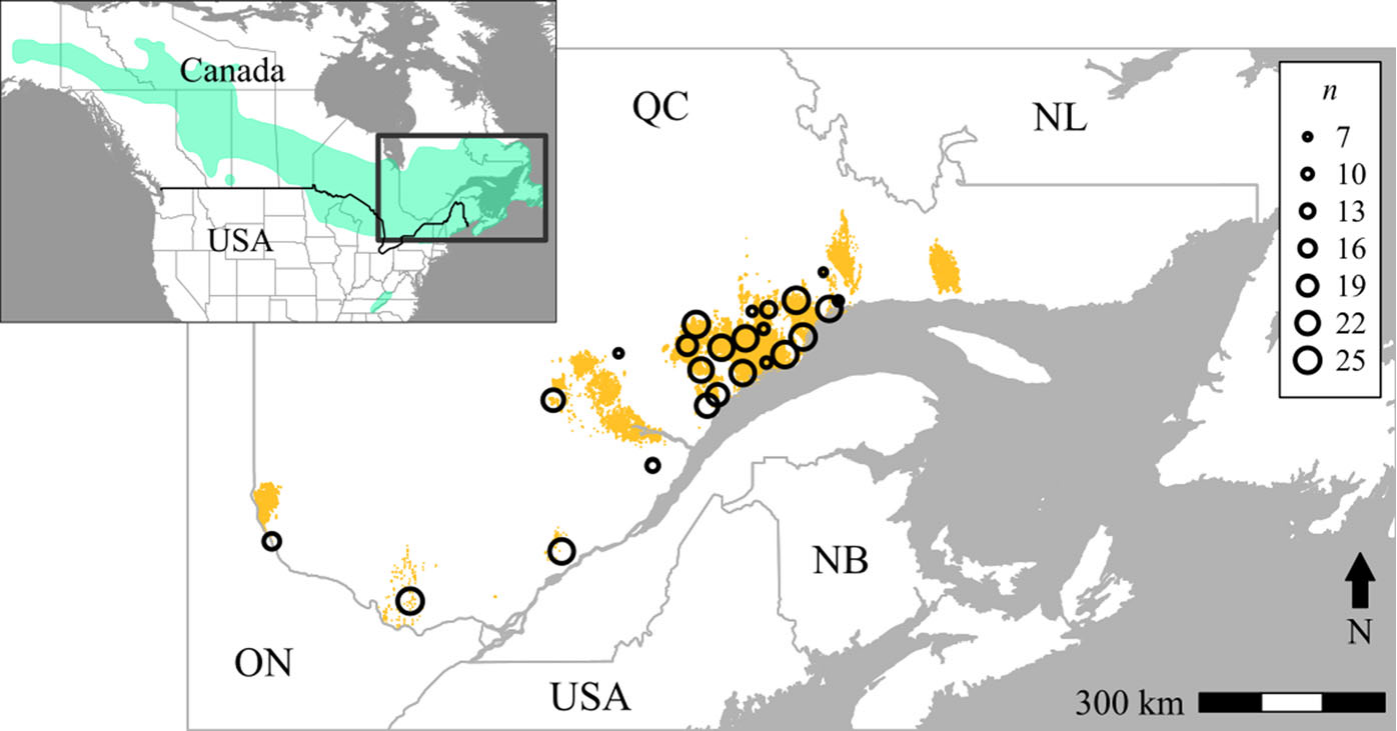
Spruce budworm distribution range, study area, sampling locations and spruce budworm defoliation areas in 2011. Sample sites are represented by points whose size is proportional to the site’s sample size (*n*). Spruce budworm distribution range. Adapted from Picq et al. (2018) is displayed in green, and defoliation areas observed the year of the sampling (2011) are represented in orange

The study was conducted in the boreal and mixed-boreal forest (Rowe 1972) in Quebec, Canada (Fig. 2), one of the areas most significantly affected by SBW outbreaks (Boulanger et al. 2012). At the time of the sampling (2012), 2.2 million ha had been affected in Quebec since the start of the outbreak in 2006 (MFFP 2020).

We used the genetic dataset previously examined in Larroque et al. (2019) for the year 2012. Briefly, 502 late-instar (L6) spruce budworm larvae and pupae were collected from 24 locations in June 2012 (Fig. 2). DNA was extracted from reared moths using Qiagen DNA Blood and Tissue kits (Venlo, Limburg, NL), prepared for Genotyping-By-Sequencing (GBS, Elshire et al. 2011) using the methods described in Brunet et al. (2017) by the Institut de Biologie Intégrative et des Systèmes (IBIS) at Université Laval (Quebec City, QC), and sequenced with an Illumina HiSeq2000 (McGill University-Génome Québec Innovation Centre, Montreal, QC). Bioinformatic processing of reads was performed using the Fast-GBS pipeline (Torkamaneh et al. 2017) resulting in 447 individuals and 3562 neutral SNPs meeting our stringent selection criteria (detailed sequencing and filtering process information can be found in Supplementary material S1).

### Possible drivers of gene flow

We tested the significance of seven landscape features that we hypothesized could influence SBW movement and gene flow: elevation, wind speed, temperature, precipitation, host cover, and defoliation (Table 1). Elevation above sea level (a.s.l) in meters was extracted using the R package *elevatr* (Hollister and Shah 2017). The highest flying moths density during dispersal events is found around 200 m above ground level (Greenbank et al. 1980), we thus extracted the raster of wind speed (m s^−1^) 200 m above ground/water level from the Global Wind Atlas (globalwindatlas.info) with a 5 km resolution. Mean July temperature (°C) and total precipitation (mm) were extracted from 289 weather stations located in the study area from the Environment and Climate Change Canada's datasets of Natural Resources Canada (NRCAN, climate-change.canada.ca). Weather station temperature and precipitation values were then interpolated onto a regular grid of a 5 km resolution using the inverse distance weight method of the R package *gstat* (Pebesma 2004). The raster layer of balsam fir (*Abies balsamea* (L.) Mill.) and white spruce (*Picea glauca* (Moench) Voss) cover (percentage of a pixel covered by the species) from 2011 was extracted from Canada’s National Forest Inventory (Beaudoin et al. 2018) at a spatial resolution of 250 m and then resampled onto a regular grid of a 5 km resolution using the R package *raster* (Hijmans 2014). Defoliation was extracted from the 2011 aerial forest survey of the MFFP and rasterized onto a regular grid of a 5 km resolution using the R package *raster* (Hijmans 2014). All predictors were represented as continuous raster layers (sensu* ResistanceGA*, a surface containing 15 or more unique values is considered as continuous, Peterman 2018) with a spatial resolution of 5 × 5 km. The spatial extent of these data was determined using the minimum convex polygon encompassing all the sampling sites with an external buffer of 75 km.

Collinearity among explanatory variables can be a severe problem when a model is trained on data from one region or time and predicted to another (Dormann et al. 2013). To reduce its negative effects, we filtered predictors based on each predictor’s Variance Inflation Factor (VIF) applying a strict threshold of 2.5 (Dormann et al. 2013). Variance inflation represents the correlation of each predictor with all others in a model and can be used to identify nonindependence among the explanatory variables (Neter et al. 1990). As the defoliation layer was the one with the highest VIF (VIF = 2.52, all others < 1.5), we subtracted the percentage of defoliation from the host cover to build only one variable of the actual host cover from which defoliated patches at the time of genetic sampling have been removed.

### Landscape genetics analyses

Parameterizing resistance surfaces in landscape genetics is not trivial (Spear et al. 2010; Zeller et al. 2012). We sought to optimally parameterize and select resistance surfaces using a genetic algorithm optimization approach as implemented in the *ResistanceGA* (Peterman 2018) R package. Genetic algorithms (Scrucca 2013) are an example of machine learning approach used to explore parameter space to find the combination of resistance surface values and transformations that maximize the statistical relationship between matrices of pairwise cost‐distances and genetic distances. The algorithm optimizes single and composite surfaces without requiring a priori resistance values based on expert opinion or ecological characteristics of the species, thus, removing potential biases introduced by inadequate knowledge of the species‐specific costs of dispersal. These surfaces are created by applying a transformation to each environmental variable that is hypothesized to influence genetic connectivity. Possible transformation functions included eight exponential-based functions, each of which is defined by two parameters, shape and maximum resistance.

Throughout the optimization process, genetic distances are regressed against resistance distances using maximum likelihood population effects (MLPE) mixed models (Clarke et al. 2002), implemented in *lme4* (Bates et al. 2015). MLPE mixed-effects models overcome the issue of nonindependence of pairwise distances by including a population covariance random-effects term that accounts for the non-independent error structure associated with pairwise distances (Clarke et al. 2002). This method has been shown to be effective at quantifying relationships between distance matrices while controlling for nonindependence in such data (Shirk et al. 2017). Potential resistance surfaces are evaluated based on their ability to model our pairwise genetic response matrix through their log-likelihood values. Resistance distances were calculated using random‐walk commute distances using *gdistance* (van Etten 2017) in R. Genetic distances were represented as a matrix of pairwise *Fst* (Weir and Cockerham 1984) values computed using the *StAMPP* package (Pembleton et al. 2013), also in R. In addition to all of the resistance surfaces built from landscape features, we examined an intercept-only model (null model), as well as a simple geographical distance surface where the resistances of all cells are set to one (*i.e.*, isolation-by-distance). In total, we compared 33 different resistance models (Supplementary material Table S2).

Following optimization of surfaces, the Akaike information criterion corrected for sample size (*AICc*) was used to select the top-ranked models (*i.e.*, *ΔAICc* < 2) of genetic distance as a function of our set of candidate models, and their absolute performances were determined using marginal *R*^*2*^_*m*_ (fixed factors) and conditional *R*^2^_*c*_ (fixed and random factors). To assess how sensitive the relative support for each hypothesis is to outliers (sites), we conducted a bootstrap resampling analysis. A subset of 75% of the populations was randomly selected 1000 times, and each time, the MLPE mixed-effects models corresponding to each of the connectivity hypotheses were fit to this subset. *AICc* was calculated for each of the 1000 refitted models. The average rank and percentage of times that each model was found the best were used as the support level. Since the optimization algorithm is a stochastic process, we conducted ten replicate runs of the whole optimization to evaluate the consistency of parameter estimates and relative relationships among resistance surfaces.

### Functional connectivity

We mapped SBW functional connectivity using randomized shortest paths algorithm (*RSP*, Saerens et al. 2009). The *RSP* algorithm incorporates elements of both least-cost paths and circuit theory, and includes a tuning parameter *θ* (0 < *θ* < 20). When *θ* is close to 0, the model is equivalent to the random‐walk commute distances and the circuit theory algorithm used by *Circuitscape* (McRae et al. 2008). When *θ* is close to 20, the model is equivalent to least-cost path models. Because our objective was to predict spruce budworm gene flow without prior knowledge of the landscape, we set *θ* to 10^–6^ to simulate a random‐walk. Connectivity was then modelled using the *RSP* algorithm implemented in the *gdistance* (van Etten 2017) R package.

We mapped SBW functional connectivity in Quebec between the north shore of the St. Lawrence river and the northern limit of the boreal forest (~ 52nd parallel, Saucier et al. 2003), with an additional buffer of 100 km. We then gathered the transformation parameters of the top-ranked landscape models (*i.e.*, *ΔAICc* < 2) of ten replicate runs, transformed the raw continuous selected surfaces into resistance surfaces using these parameters, and calculated the average resistance of each pixel to produce a map of average resistance. Following Koen et al. (2014), we regularly placed 50,000 nodes (*i.e.*, source and destination sites for the *RSP* algorithm) within the 100 km buffer of the average resistance map and simulated the *RSP* passing between all pairs of nodes, summed the total number of passages in each cell, and then removed the buffer to eliminate any edge effects (Koen et al. 2010). The resulting map illustrates the most likely paths of the spread of the outbreak, conceptually similar to the mapping of current flow in circuit theory (McRae et al. 2008).

### Predicted functional connectivity

As identifying future areas of high connectivity is critical for large‐scale early forest management strategies, we wanted to predict potential connectivity at the beginning of the next anticipated outbreak. To make such a forecast, we made the following assumptions: (1) the most important selected landscape variables and their resistance values will apply equally in the future. However, the role of natural selection on dispersal in response to landscape resistance and its effects on population connectivity over longer time scales remains uncertain (e.g., Lowe and McPeek 2014); (2) because the current outbreak started in approximately 2006, and we know that SBW outbreaks have a periodicity of ~ 35 years (Royama 1984), we expect the next outbreak to commence at or around 2040. Therefore, we used the weather prediction of the high-emission scenario [Representative Concentration Pathway (RCP) 8.5] to predict the future climate conditions and associated SBW connectivity in the year 2040.

Given our model selection results (see “Results” section), we used host cover and precipitation level to predict future connectivity. Because the speed of migration of forest tree species is 10–100 times slower than that predicted for the shift in climatic niches (Natural Resources Canada 2016), we assume that tree host spatial distribution will remain constant for our forecast in 2040. We used the predicted precipitation of July 2040 (Natural Resources Canada, climate-change.canada.ca). We then followed the same procedure explained above: we used the transformation parameters of the top-ranked landscape models of the ten replicate runs, transformed the raw continuous surfaces to resistance surfaces using these parameters and calculated the average resistance of each pixel. We then simulated the *RSP* passing between all pairs of the 50,000 nodes and summed the total number of passages in each cell. The resulting map illustrates potential paths of the spread of the next outbreak in 2040.

## Results

Final models selected from our optimization and model selection procedure were consistent over the ten replicate runs: the *precipitation* model was selected in the top-ranked models (*i.e.*, *ΔAICc* < 2) in nine replications, and the *precipitation* + *host cover* in three replications (Table 2). When the composite surfaces *precipitation* + *host cover* was selected, *precipitation* and *host cover* costs contributed 31% (± 2) and 69% (± 2), respectively, to the total cost of traveling through a cell.

**Table 2:** Averaged values of the top-ranked landscape models for the ten replications

	*n*	*AICc*	*R*^*2*^_*m*_	*R*^*2*^_*c*_	Bootstrap %	Rank
*Precipitation*	9	− 2603.97 ± 1.52	0.42 ± 0.02	0.75 ± 0.01	57.13 ± 5.99	1.11 ± 0.33
*Precipitation* + *host cover*	3	− 2602.68 ± 0.32	0.51 ± 0.02	0.75 ± 0.01	40.1 ± 25.46	1.33 ± 0.58

In the table, *n* is the number of times each surface has been selected as the top-ranked model; *AICc* is the *AIC* value of the model corrected for the number of parameters optimized and the sample size; *R*^*2*^_*m*_ and *R*^*2*^_*c*_ are the marginal and the conditional *R*^*2*^; “Bootstrap %” is the average frequency of the model reaching the top rank during the bootstrap procedure, “Rank” is the average rank achieved by the model during the bootstrap procedure. The *precipitation* model has been selected 9 times and the *precipitation* + *host cover* model 3 times

Over the ten replicate runs, both models were consistently ranked highly: rank = 1.11 ± 0.33 and 1.33 ± 0.58, respectively. Models using *precipitation* and *precipitation* + *host cover* as predictors achieved a marginal *R*^*2*^_*m*_ of 0.42 ± 0.02 and 0.51 ± 0.02 (conditional *R*^*2*^_*c*_ = 0.75 ± 0.01 for both models), demonstrating good performance. Neither the null model (averaged *ΔAICc* = 8.06 ± 1.27) nor the isolation-by-distance model (averaged *ΔAICc* = 4.08 ± 1.27) were selected.

**Fig. 3:**
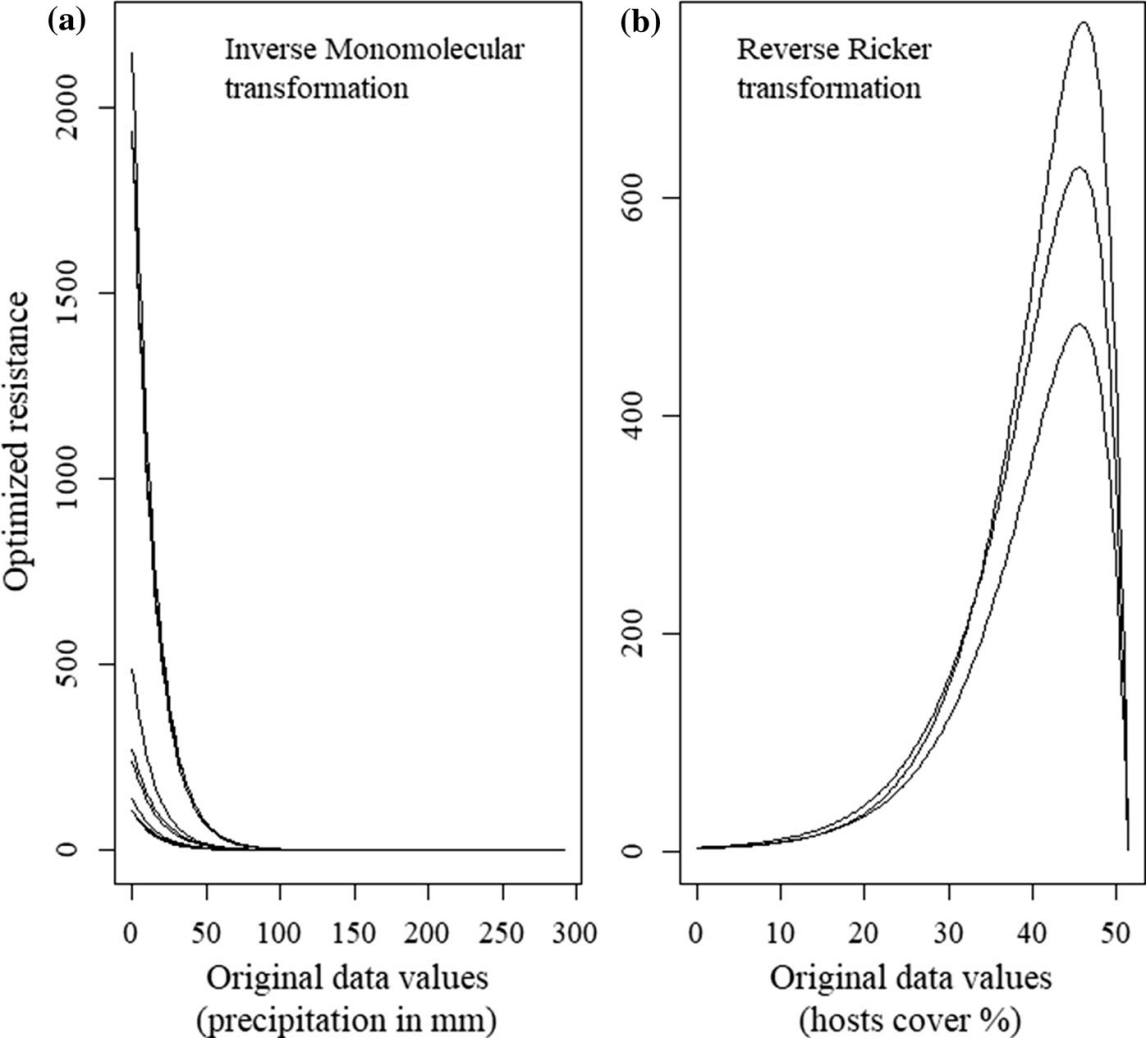
Surface optimization results for the two environmental variables selected in the top-ranked landscape models (*i.e.*, *ΔAICc* < 2) of the ten replicated runs: **a** precipitation (inverse monomolecular transformation, *n* = 9), and **b** host cover (Reverse Ricker transformation, *n* = 3)

Even if the maximum resistance selected by the genetic algorithm for the top-ranked models, *i.e.*, for the *precipitation* and *host cover* layers varied (Fig. 3), the transformation functions selected were consistent over the replicate runs. The algorithm identified a fast decrease in the cost of movement with increasing precipitation levels with a minimum between 25 and 75 mm (Fig. 3a). A unimodal transformation with a maximum cost at a cover value of 45% was fitted for the host cover surface. From 0 to 20%, the resistance is minimal. Then above 20%, the resistance increases to reach its maximum value for a host cover of 45%. Finally, above 45%, the resistance declines steeply until it reaches its minimum level (Fig. 3b). The low resistance for cover > 45% might be an artifact of the optimization due to the very low availability (~ 0.12%) of such high cover in the study area.

**Fig. 4:**
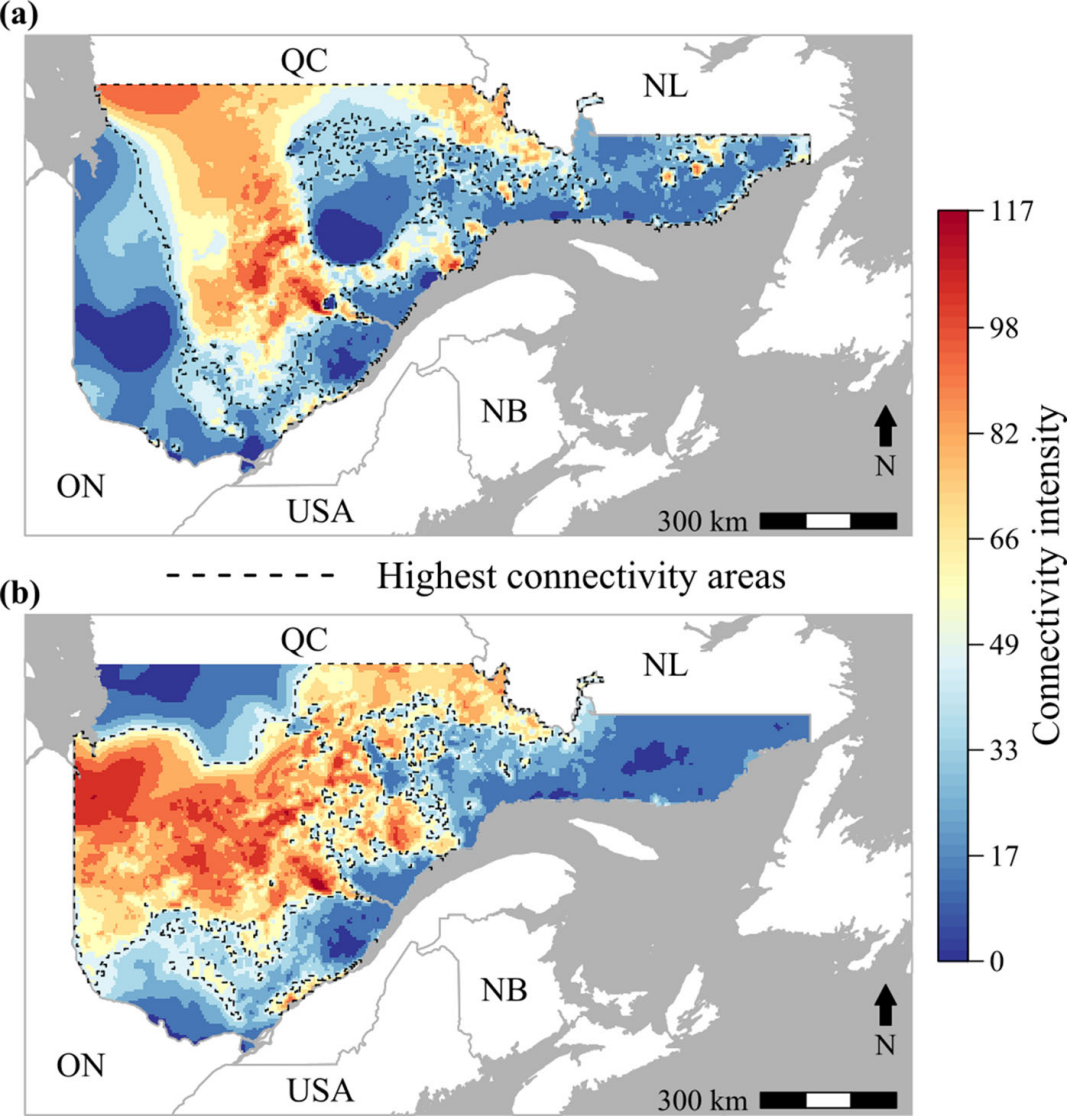
SBW genetic connectivity illustration. **a** Randomized shortest paths (*RSP*) predictions of SBW dispersal in Quebec based on the averaged top-ranked landscape models of the ten replications. Color gradient is based on cumulative values of *RSP*s for 50,000 pairs of origin–destination nodes, representing the number of passages for each grid cell. Dashed contour lines indicate connectivity corridors based on the *RSP* map by keeping only the 50% maximum connectivity values. **b** Future *RSP* predictions of the 2040 SBW dispersal. Dashed contour lines indicate future connectivity corridors based on the *RSP* map by keeping only the 50% maximum 2040 connectivity values

The current map illustrating the present functional connectivity in Quebec showed one large high connectivity area (Fig. 4a) surrounded by two low-connectivity areas to the East and West. By keeping only the highest 50% connectivity values, we found that the western part of Quebec could potentially act as a barrier with Ontario (Fig. 4a). In contrast, in the eastern part of Quebec, multiple high connectivity patches could act as stepping-stones, facilitating dispersal (Fig. 4a).

**Fig. 5:**
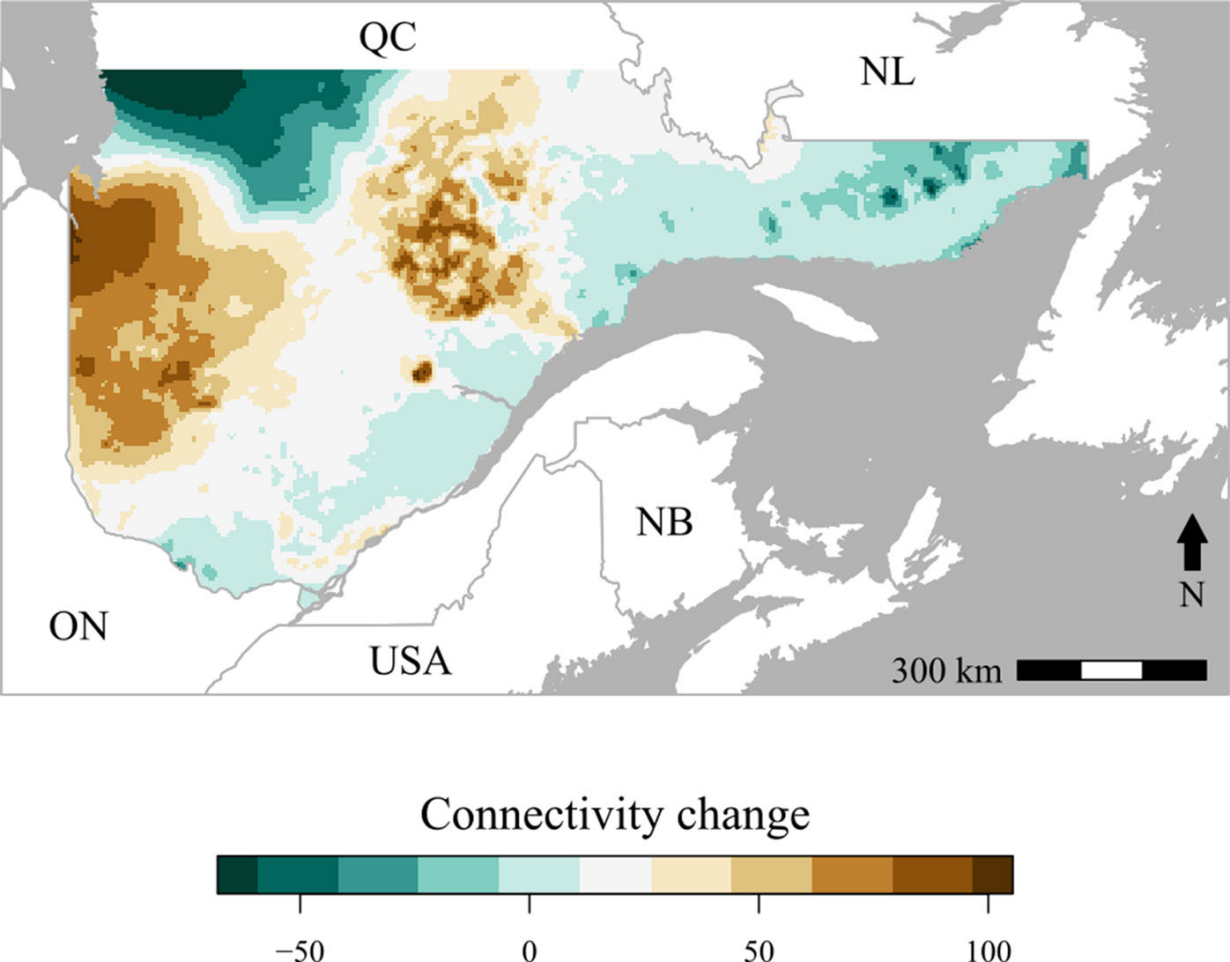
Changes in SBW genetic connectivity. Difference between the future 2040 predicted connectivity and the present predicted connectivity. Green colors indicate a decrease in connectivity while brown colors indicate an increase in connectivity

Predicted connectivity for 2040 (Fig. 4b) showed a region of high connectivity that included two thirds of Quebec and is connected to the border with Ontario. In contrast, the eastern part of Quebec is disconnected and did not show any high connectivity areas (Fig. 4b). Future connectivity is expected to increase in the western part of Quebec and decrease in the eastern part relative to the present predicted connectivity (Fig. 5).

## Discussion

### Landscape determinants of population connectivity

As expected, forest composition, specifically the percentage of cover of white spruce and balsam fir affects SBW dispersal, with the lowest cover showing the lowest resistance. Host cover likely does not affect dispersal directly. Rather, the abundance and quality of hosts affect local population densities which can increase outbound propagule pressure, as well as the decision to disperse (Dwyer and Morris 2006); low resource quality and availability have been shown to encourage dispersal in Lepidopterans (Rhainds et al. 2002). Morris and Mott (1963) showed that SBW dispersal is density-dependent such that dispersal rates increase with increased defoliation as the trees became less attractive as oviposition-sites and because of food depletion in increased competition (Régnière and Nealis 2007; Van Hezewijk et al. 2018). In addition to food shortage, crowding is another proximate cause of pest insect dispersal because of its negative effect on insect fitness (Mazzi and Dorn 2012). Previous work has also shown that SBW moths tend to emigrate from high-density populations (Greenbank 1963; Régnière and Nealis 2019). SBW dispersal might thus be promoted by low host cover as SBW individuals seek to escape low food resources and high population densities during outbreaks. In contrast, areas with high levels of host cover can act as demographic sinks as the good conditions might dissuade individuals from leaving. However, it should be noted that host cover was always associated with precipitation, which also seems to play an important role in determining SBW connectivity.

Precipitation was selected in all the top-ranked models across the ten replicated runs with the same transformation consistently identified (Fig. 3a). Low levels of precipitation, *i.e.*, under a 25–75 mm threshold, were found to strongly decrease connectivity, whereas areas with greater precipitation showed no resistance at all. Precipitation can act both on dispersal movement, and the probability of success of the settlement phase through survival. The mechanism underlying the relationship between precipitation and dispersal per se is not clear. Dickison et al. (1986) showed that dispersal activity was greater on nights of widespread and heavy rain, and Greenbank et al. (1980) showed that SBW can fly through light or moderate rain but heavy rain limits take-offs. However, Sturtevant et al. (2013) showed that above an unknown threshold, precipitation may cause forced descent and affect landing success, decreasing the frequency of long-dispersal events. The link between precipitation and SBW survival is clearer, while Moise et al. (2019) showed that precipitation level seems to have a limited effect on SBW performance, increasing precipitation had positive effects on tree growth during SBW outbreaks (Fierravanti et al. 2015), potentially increasing early instar larval survival (Pureswaran et al. 2018b). This effect has been confirmed by a meta-analysis, Koricheva et al. (1998) showed that the water stress effect was insect feeding guild-dependent, and that chewing insects generally underwent performance reduction on stressed slow-growing plants compared to stressed fast growers. Precipitation levels > 75 mm might thus favor SBW survival and long-term connectivity in Quebec.

We did not find any effect of elevation or temperature. With a maximum elevation of 950 m a.s.l and less than 16% above 500 m a.s.l, the topography may not constitute an impassable barrier for the SBW which is able to fly at altitude exceeding 1000 m (Sturtevant et al. 2013), and that could explain why we did not find any elevation effect on the genetic structure. With an averaged *ΔAICc* = 6.21 ± 1.29, the absence of an effect of temperature is more surprising. Several studies have shown that temperature is important to SBW phenology (Régnière and You 1991), flight activity (Sanders et al. 1978; Greenbank et al. 1980), flight altitude (Régnière et al. 2019), and survival through the phenological synchrony with its hosts (Pureswaran et al. 2018a). However, our spatial information on temperature showed a low degree of variability (mean July temperature = 17.63 ± 1.96 °C) over the study area. When an environmental feature shows little spatial variation, a landscape genetics approach may fail to identify such a feature as the one driving connectivity (Short Bull et al. 2011; Cushman et al. 2013), even if it is relevant over larger spatial scales. Thus, temperature may be an important factor for population connectivity, but at a much larger spatial scale than the scale we examined here.

Even if the SBW is a strong flyer able to fly upwards of 50 km to exhaustion without wind assistance (Sturtevant et al. 2013), it has been shown that as for most forest pests, wind has a strong influence of SBW long distance dispersal (up to 450 km, Greenbank et al. 1980). However, we found no evidence for an effect of wind speed effect in our model selection. With an averaged *ΔAICc* = 6.28 ± 1.27 over the 10 replicated runs, the effect of wind speed seems negligible in comparison to the effect of precipitation. But, by considering only wind speed, we assumed isotropic resistance to movement, *i.e.*, the resistance between two sites is the same regardless of the direction being travelled. However, for wind‐assisted species, dispersal can be highly directional and this asymmetric process can play a significant role in shaping spatial genetic structure (Holderegger and Gugerli 2012). While it is technically feasible and biologically pertinent to compute asymmetric effective distances (e.g., Wang 2020) and asymmetric gene flow estimates (e.g., Sundqvist et al. 2016), to our knowledge, no effective and well-tested tools exist to make such comparisons. We identify the development of methods allowing to consider both asymmetric dispersal rate and cost as a key area for the future development of landscape genetics.

### Present and future connectivity

Using our connectivity model, we found that while two parts of Quebec showed a very low connectivity, overall, there seem to be few constraints to SBW movement at the scale of the province of Quebec. It is too late to stop the progression of the present outbreak in Quebec, the defoliation has grown by 40% between 2019 and 2020 (MFFP 2020). However, our model could be used to forecast connectivity between affected and unaffected stands and to prioritize well-connected, but not yet attacked, stands for monitoring, and potential pre-emptive treatment such as spraying or harvest. Additionally, it will be interesting to validate our connectivity map with field observations of the flying events of SBW. This approach has been used for large species such as the puma (*Puma concolor*, Zeller et al. 2018) but could be challenging especially for flying insects (Osborne et al. 2002). However, spruce budworm monitoring is currently undertaken using pheromone-baited moth traps (Carleton et al. 2020) and L2 collection from branch samples (Johns et al. 2019), and tracking dispersing flying moths has been made possible using weather surveillance radar (e.g., Boulanger et al. 2017). Quantifying the proportion of mass dispersal events occurring in and out of the high connectivity areas could help to improve connectivity estimations.

Our model predictions suggest that while connectivity is generally high over much of Quebec, it may increase significantly by the next outbreak (~ 2040). During the twentieth century, the range of the SBW has moved northward (Navarro et al. 2018). With climate change, the northern boreal forest could become more vulnerable to outbreaks as a result of a better phenological synchrony with the SBW secondary host, the black spruce (Pureswaran et al. 2018a), which is expected to increase the frequency and severity of future outbreaks (Navarro et al. 2018). Our analyses also suggest that future northward outbreak spread might be facilitated by increased precipitation, which may further increase SBW functional connectivity. Only the most eastern part of Quebec is expected to see a reduction in connectivity, which may reduce the risk of spread to other eastern provinces. However, spatial climate modeling is an ongoing task (McKenney et al. 2011), as source datasets change in coverage and quality and as new applications and methods evolve, these predictions might change.

## Conclusion

Despite its importance to outbreak dynamics, the role of landscape heterogeneity in shaping spruce budworm connectivity has not been previously examined. We found that a combination of precipitation and host cover constitute the best predictor of SBW genetic connectivity in Quebec in the current outbreak. Although we did not identify any discrete barriers to dispersal, some low connectivity areas might constrain the dispersal in some areas, in the current and future outbreaks, that should in turn be intensively monitored and be the location of management measures such as the Early Intervention Strategy (Johns et al. 2019; MacLean et al. 2019).

Consideration of the demographic context is particularly important for cyclic irruptive species when evaluating the role of spatial heterogeneity in population connectivity using landscape genetics (Fig. 1). Spatial patterns of genetic diversity are the result of multiple factors that interact through space and time, and the ability to distinguish their relative contribution will vary depending on the characteristics of the species being studied. For example, landscape genetics approaches may be sensitive to species‐specific factors such as effective population size, dispersal capacity, and landscape heterogeneity. We are confident in our results as we sampled early in the outbreak before the decline in spatial genetic structure through time (Larroque et al. 2019), *i.e.*, before the effect of the landscape has been blurred by mass dispersal events. Further exploration of the temporal dynamics of spatial genetic structure during population outbreaks under different contexts remains a promising avenue for future research. Simulation‐based approaches using spatially explicit demo‐genetic models (e.g., Nemo, Guillaume and Rougemont 2006) hold great promise to isolate and quantify the relative effects of these different factors on the development of spatial genetic structure.

Despite being the area the most susceptible to SBW outbreaks (Blais 1983), the spatial extent of our study (Quebec) represents only a portion of the SBW’s continental range. However, connectivity is a scale-dependent process, and estimates through landscape genetic analyses are sensitive to the spatial scale of measurement and analysis (Anderson et al. 2010; Cushman and Landguth 2010). Over the full SBW range, Lumley et al. (2020) identified three spatial genetic clusters: Western (Alaska, Yukon), Central (southeast Yukon to Manitoba), and Eastern (Ontario to Atlantic) clusters. These three geographical areas display different key environmental and structural landscape elements such as mountain ranges between the Western and Central subpopulations, host distribution differences (e.g., the balsam fir distribution does not extend up to the full range of spruce budworm), or lower temperature in western Canada that could decrease overwintering survival. SBW dispersal is therefore likely to be impacted by variables different from those identified in Quebec and these clusters with different latitudes might also react differently to global change (Pureswaran et al. 2018b; Lehmann et al. 2020). Analysis of connectivity in multiple landscapes would provide more robust results for management applications across geographic regions (Short Bull et al. 2011; Larroque et al. 2016). Replication of our approach to other distinct regions within the SBW geographic range would further improve our understanding of these complex relationships between dispersal, landscape structure, and outbreak dynamics.

## Supplementary Information

Below is the link to the electronic supplementary material.

Supplementary file1 (DOCX 98 kb)

## Data Availability

One Genalex file representing the filtered SNPs data set used for analyses and the reference genome version used in this manuscript are available at the Dryad Digital Repository: https://doi.org/10.5061/dryad.1vr6g3f.
