# Supplementary material for: Quantifying and predicting population connectivity of an outbreaking forest insect pest
Source: Landsc Ecol. 2021 Dec 23;37(3):763–78. doi: 10.1007/s10980-021-01382-9 (PMC8897358; doi:10.1007/s10980-021-01382-9)
Supplement: Supplementary file 1 — Supplementary file1 (DOCX 98 kb) [file 10980_2021_1382_MOESM1_ESM.docx]

**Supplementary material for**

**Quantifying and predicting population connectivity of an outbreaking forest insect pest**

**S1 Sequencing and filtering**

All DNA samples were prepared for Genotyping-By-Sequencing (GBS, Elshire et al. 2011) using the methods described in Brunet et al. (2017). *PstI-MspI* GBS libraries (96 plex) were prepared by the Institut de Biologie Intégrative et des Systèmes (IBIS) at Université Laval (Quebec City, QC) using the protocol of Poland et al. (2012). Following final amplification of the pooled, adapter-ligated restriction fragments, 600 ng of each amplified library was normalized to remove the repetitive fraction by treatment with duplex-specific nuclease (Zhulidov et al. 2004). Finally, an additional PCR step using a selective reverse primer extending a single base (C) into the insert past the 3′ restriction site was used to selectively amplify one-quarter of the total number of fragments, thereby increasing the read depth of sequenced fragments (Sonah et al. 2013). Single-end sequencing (100 bp reads) of these libraries was then performed with an Illumina HiSeq2000 (McGill University-Génome Québec Innovation Centre, Montreal, QC).

Bioinformatic processing of reads was performed using the Fast-GBS pipeline (Torkamaneh et al. 2017). The Fast-GBS pipeline includes demultiplexing, trimming, mapping, and variant calling steps to process Genotyping-By-Sequencing samples and provide highly accurate genotyping. This pipeline has been shown to yield the highest accuracy compared to the other pipelines (Torkamaneh et al. 2016). After demultiplexing and adapter trimming, reads less than 50 bp were discarded. A total of 4.162 gigabases of reads were aligned on the spruce budworm reference genome (bw6 version, manuscript in preparation, for questions about the genome assembly used as reference in this study, please contact M. Cusson (michel.cusson@canada.ca)). Alignment of 3.141 gigabases of reads was performed using the Burrows–Wheeler Aligner (BWA, Li and Durbin 2010) with the *mem* algorithm, and yielded a 75% mapping success. SNP calls were made as part of the Fast-GBS pipeline using Platypus (v0.8.1, Rimmer et al. 2014). Minimum read depth was set to eight reads. Only bi-allelic SNPs, with a maximum of 50% missing genotypes throughout all samples were retained. Individuals with more than 50% missing genotypes were removed. Finally, as some methods cannot handle missing data, missing genotypes were imputed using the software BEAGLE (v3.3.2, Browning and Browning 2016) which replaces missing genotypes with the most frequently observed genotype associated with proximal SNP loci. Variants with *R*² (*i.e.*, imputation accuracy) < 0.4 were removed as advised by Browning and Browning (2016).

SNPs with a minor allele frequency (MAF) < 5% were removed to exclude putative sequencing errors and keep only the most informative SNPs (Marees et al. 2018) Similarly, SNPs in high linkage disequilibrium (LD) at a threshold of *r²* ≥ 0.2 were discarded to remove highly correlated variants that add minimal extra information and could overly influence some methods (Price et al. 2008; Zou et al. 2010), using the *snpgdsLDpruning* function of the *SNPRelate* package (Zheng et al. 2012) in R (R Core Team 2017). SNPs showing deviation from expected Hardy-Weinberg equilibrium (HWE) in more than 15% of the sites were also removed as deviation from HWE can be indicative of genotyping errors (Anderson et al. 2010), null alleles (Brookfield 1996) or selection (Wittke-Thompson et al. 2005). HWE was calculated using the *hw.tes*t function of the *pegas* package in R (Paradis 2010) and using a Bonferroni correction for multiple comparisons. Finally, as our aim was to quantify the neutral evolutionary process of gene flow, we removed all SNPs identified as potentially under selection (Beaumont and Nichols 1996) using the *pcadapt* function of the *pcadapt* package in R (Luu et al. 2017).

*References*

Anderson CA, Pettersson FH, Clarke GM, Cardon LR, Morris AP, Zondervan KT (2010) Data quality control in genetic case-control association studies. Nature protocols 5(9):1564-1573

Beaumont MA, Nichols RA (1996) Evaluating loci for use in the genetic analysis of population structure. Proceedings of the Royal Society of London. Series B: Biological Sciences 263(1377):1619-1626

Brookfield JFY (1996) A simple new method for estimating null allele frequency from heterozygote deficiency. Mol Ecol 5(3):453-455

Browning BL, Browning SR (2016) Genotype imputation with millions of reference samples. The American Journal of Human Genetics 98(1):116-126

Brunet BMT, Blackburn GS, Muirhead K et al (2017) Two's company, three's a crowd: new insights on spruce budworm species boundaries using genotyping-by-sequencing in an integrative species assessment (Lepidoptera: Tortricidae). Syst Entomol 42(2):317-328

Elshire RJ, Glaubitz JC, Sun Q et al (2011) A robust, simple genotyping-by-sequencing (GBS) approach for high diversity species. PLoS ONE 6(5):e19379

Li H, Durbin R (2010) Fast and accurate long-read alignment with Burrows-Wheeler transform. Bioinformatics 26(5):589-595

Luu K, Bazin E, Blum MGB (2017) pcadapt: an R package to perform genome scans for selection based on principal component analysis. Molecular Ecology Resources 17(1):67-77

Marees AT, de Kluiver H, Stringer S et al (2018) A tutorial on conducting genome-wide association studies: Quality control and statistical analysis. International Journal of Methods in Psychiatric Research 27(2):e1608

Paradis E (2010) pegas: an R package for population genetics with an integrated-modular approach. Bioinformatics 26(3):419-20

Poland JA, Brown PJ, Sorrells ME, Jannink J-L (2012) Development of high-density genetic maps for barley and wheat using a novel two-enzyme genotyping-by-sequencing approach. PLoS ONE 7(2):e32253

Price AL, Weale ME, Patterson N et al (2008) Long-range LD can confound genome scans in admixed populations. The American Journal of Human Genetics 83(1):132-135

R Core Team (2017) R: A Language and Environment for Statistical Computing. R Foundation for Statistical Computing, Vienna, Austria,

Rimmer A, Phan H, Mathieson I et al (2014) Integrating mapping-, assembly- and haplotype-based approaches for calling variants in clinical sequencing applications. Nat. Genet. 46(8):912-918

Sonah H, Bastien M, Iquira E et al (2013) An improved genotyping by sequencing (GBS) approach offering increased versatility and efficiency of SNP discovery and genotyping. PLoS ONE 8(1):e54603

Torkamaneh D, Laroche J, Bastien M, Abed A, Belzile F (2017) Fast-GBS: a new pipeline for the efficient and highly accurate calling of SNPs from genotyping-by-sequencing data. BMC Bioinformatics 18:5

Torkamaneh D, Laroche J, Belzile F (2016) Genome-wide SNP calling from genotyping by sequencing (GBS) data: a comparison of seven pipelines and two sequencing technologies. PLoS ONE 11(8):e0161333

Wittke-Thompson JK, Pluzhnikov A, Cox NJ (2005) Rational inferences about departures from Hardy-Weinberg equilibrium. Am. J. Hum. Genet. 76(6):967-86

Zheng X, Levine D, Shen J, Gogarten SM, Laurie C, Weir BS (2012) A high-performance computing toolset for relatedness and principal component analysis of SNP data. Bioinformatics 28(24):3326-3328

Zhulidov PA, Bogdanova EA, Shcheglov AS et al (2004) Simple cDNA normalization using kamchatka crab duplex-specific nuclease. Nucleic Acids Res 32(3):e37

Zou F, Lee S, Knowles MR, Wright FA (2010) Quantification of population structure using correlated SNPs by shrinkage principal components. Human heredity 70(1):9-22

**Table S2** Model selection and bootstrap analysis results sorted by *AICc* for the ten replicated runs. *AICc* is the *AIC* value of the model corrected for the number of parameters *k* and the sample size; ΔAICc is the difference between the *AICc* of the model and the minimum *AICc* across all models; *ω_AICc_* is the Akaike weight of the model; *R^2^_m_* and *R^2^_c_* are the marginal and the conditional *R^2^*; *LL* is the log-likelihood; “B%” is the average frequency of the model reaching the top rank during the bootstrap procedure, “Rank” is the average rank achieved by the model during the bootstrap procedure.

| Surface | *k* | *AIC* | *AICc* | *R^2^_m_* | *R^2^_c_* | *LL* | *ΔAICc* | *ω_AICc_* | Rank | B% |
| --- | --- | --- | --- | --- | --- | --- | --- | --- | --- | --- |
| replicate 1 | | | | | | | | | | |
| **precip** | **4** | **-2605.168** | **-2603.062** | **0.424** | **0.752** | **1306.584** | **0.000** | **0.553** | **3.146** | **54.1** |
| precip+hosts | 7 | -2612.922 | -2599.922 | 0.405 | 0.702 | 1310.461 | 3.140 | 0.115 | 3.921 | 21.1 |
| Distance | 2 | -2596.492 | -2599.921 | 0.027 | 0.684 | 1302.246 | 3.141 | 0.115 | 5.232 | 0.4 |
| hosts | 4 | -2601.380 | -2599.275 | 0.095 | 0.681 | 1304.690 | 3.788 | 0.083 | 4.02 | 21.4 |
| wind | 4 | -2599.833 | -2597.728 | 0.111 | 0.713 | 1303.916 | 5.335 | 0.038 | 5.965 | 2.1 |
| temp | 4 | -2599.811 | -2597.706 | 0.113 | 0.685 | 1303.906 | 5.356 | 0.038 | 5.075 | 0.2 |
| elev | 4 | -2598.652 | -2596.547 | 0.081 | 0.700 | 1303.326 | 6.515 | 0.021 | 6.909 | 0.7 |
| NULL | 1 | -2592.124 | -2595.942 | 0.000 | 0.671 | 1299.062 | 7.120 | 0.016 | - | - |
| precip+elev | 7 | -2608.548 | -2595.548 | 0.332 | 0.705 | 1308.274 | 7.514 | 0.013 | 7.328 | 0 |
| wind+precip | 7 | -2605.331 | -2592.331 | 0.437 | 0.755 | 1306.666 | 10.731 | 0.003 | 9.591 | 0 |
| temp+precip | 7 | -2604.787 | -2591.787 | 0.358 | 0.738 | 1306.393 | 11.275 | 0.002 | 10.193 | 0 |
| temp+hosts | 7 | -2602.977 | -2589.977 | 0.069 | 0.672 | 1305.488 | 13.086 | 0.001 | 10.428 | 0 |
| wind+hosts | 7 | -2601.486 | -2588.486 | 0.080 | 0.683 | 1304.743 | 14.576 | 0.000 | 12.279 | 0 |
| elev+hosts | 7 | -2601.338 | -2588.338 | 0.098 | 0.681 | 1304.669 | 14.725 | 0.000 | 11.953 | 0 |
| temp+elev | 7 | -2601.160 | -2588.160 | 0.063 | 0.678 | 1304.580 | 14.902 | 0.000 | 12.832 | 0 |
| wind+elev | 7 | -2600.692 | -2587.692 | 0.089 | 0.703 | 1304.346 | 15.370 | 0.000 | 13.812 | 0 |
| wind+temp | 7 | -2600.504 | -2587.504 | 0.087 | 0.689 | 1304.252 | 15.559 | 0.000 | 13.488 | 0 |
| temp+precip+hosts | 10 | -2609.829 | -2580.905 | 0.386 | 0.719 | 1308.914 | 22.157 | 0.000 | 18.775 | 0 |
| wind+precip+elev | 10 | -2608.767 | -2579.844 | 0.315 | 0.696 | 1308.383 | 23.219 | 0.000 | 19.098 | 0 |
| precip+elev+hosts | 10 | -2604.853 | -2575.930 | 0.122 | 0.663 | 1306.427 | 27.132 | 0.000 | 20.123 | 0 |
| wind+precip+hosts | 10 | -2603.060 | -2574.137 | 0.088 | 0.675 | 1305.530 | 28.926 | 0.000 | 21.323 | 0 |
| temp+precip+elev | 10 | -2602.987 | -2574.064 | 0.142 | 0.695 | 1305.494 | 28.998 | 0.000 | 21.762 | 0 |
| wind+temp+hosts | 10 | -2602.941 | -2574.018 | 0.068 | 0.672 | 1305.470 | 29.045 | 0.000 | 21.453 | 0 |
| temp+elev+hosts | 10 | -2602.906 | -2573.983 | 0.069 | 0.672 | 1305.453 | 29.080 | 0.000 | 21.741 | 0 |
| wind+temp+precip | 10 | -2602.407 | -2573.484 | 0.151 | 0.694 | 1305.203 | 29.578 | 0.000 | 22.392 | 0 |
| wind+temp+elev | 10 | -2601.408 | -2572.485 | 0.079 | 0.691 | 1304.704 | 30.577 | 0.000 | 24.161 | 0 |
| wind+elev+hosts | 10 | -2600.512 | -2571.589 | 0.097 | 0.695 | 1304.256 | 31.474 | 0.000 | 24 | 0 |
| wind+temp+precip+hosts | 13 | -2603.905 | -2549.505 | 0.117 | 0.677 | 1305.952 | 53.557 | 0.000 | 27.657 | 0 |
| temp+precip+elev+hosts | 13 | -2603.489 | -2549.089 | 0.130 | 0.685 | 1305.745 | 53.973 | 0.000 | 28.523 | 0 |
| wind+precip+elev+hosts | 13 | -2603.423 | -2549.023 | 0.127 | 0.686 | 1305.712 | 54.039 | 0.000 | 28.714 | 0 |
| wind+temp+elev+hosts | 13 | -2602.478 | -2548.078 | 0.072 | 0.678 | 1305.239 | 54.984 | 0.000 | 29.723 | 0 |
| wind+temp+precip+elev | 13 | -2602.175 | -2547.775 | 0.093 | 0.693 | 1305.088 | 55.287 | 0.000 | 30.383 | 0 |
| wind+temp+precip+elev+hosts | 16 | -2603.648 | -2501.933 | 0.104 | 0.675 | 1305.824 | 101.129 | 0.000 | 32 | 0 |

| Surface | *k* | *AIC* | *AICc* | *R^2^_m_* | *R^2^_c_* | *LL* | *ΔAICc* | *ω_AICc_* | Rank | B% |
| --- | --- | --- | --- | --- | --- | --- | --- | --- | --- | --- |
| replicate 2 | | | | | | | | | | |
| **precip** | **4** | **-2607.624** | **-2605.519** | **0.397** | **0.739** | **1307.812** | **0.000** | **0.782** | **2.573** | **64.7** |
| precip+hosts | 7 | -2613.800 | -2600.800 | 0.511 | 0.758 | 1310.900 | 4.719 | 0.074 | 4.188 | 10.1 |
| Distance | 2 | -2596.492 | -2599.921 | 0.027 | 0.684 | 1302.246 | 5.598 | 0.048 | 5.347 | 0.1 |
| hosts | 4 | -2601.393 | -2599.288 | 0.099 | 0.681 | 1304.697 | 6.231 | 0.035 | 4.207 | 22.6 |
| wind | 4 | -2599.834 | -2597.728 | 0.112 | 0.713 | 1303.917 | 7.790 | 0.016 | 6.32 | 2.1 |
| temp | 4 | -2599.442 | -2597.337 | 0.112 | 0.681 | 1303.721 | 8.182 | 0.013 | 5.742 | 0.2 |
| precip+elev | 7 | -2609.918 | -2596.918 | 0.476 | 0.764 | 1308.959 | 8.601 | 0.011 | 6.922 | 0 |
| Elev | 4 | -2598.664 | -2596.559 | 0.070 | 0.695 | 1303.332 | 8.960 | 0.009 | 7.164 | 0.2 |
| NULL | 1 | -2592.124 | -2595.942 | 0.000 | 0.671 | 1299.062 | 9.576 | 0.007 | - | - |
| temp+precip | 7 | -2607.433 | -2594.433 | 0.418 | 0.751 | 1307.717 | 11.085 | 0.003 | 9.538 | 0 |
| wind+precip | 7 | -2606.988 | -2593.988 | 0.269 | 0.683 | 1307.494 | 11.531 | 0.002 | 9.014 | 0 |
| temp+hosts | 7 | -2602.445 | -2589.445 | 0.061 | 0.670 | 1305.223 | 16.074 | 0.000 | 10.908 | 0 |
| temp+elev | 7 | -2601.747 | -2588.747 | 0.074 | 0.679 | 1304.874 | 16.771 | 0.000 | 12.109 | 0 |
| wind+hosts | 7 | -2601.395 | -2588.395 | 0.078 | 0.685 | 1304.698 | 17.123 | 0.000 | 12.343 | 0 |
| wind+elev | 7 | -2600.688 | -2587.688 | 0.085 | 0.701 | 1304.344 | 17.831 | 0.000 | 14.004 | 0 |
| wind+temp | 7 | -2600.634 | -2587.634 | 0.084 | 0.684 | 1304.317 | 17.885 | 0.000 | 13.673 | 0 |
| elev+hosts | 7 | -2600.527 | -2587.527 | 0.098 | 0.681 | 1304.264 | 17.992 | 0.000 | 12.439 | 0 |
| wind+precip+hosts | 10 | -2612.501 | -2583.578 | 0.365 | 0.692 | 1310.250 | 21.941 | 0.000 | 17.683 | 0 |
| temp+precip+elev | 10 | -2611.763 | -2582.840 | 0.427 | 0.745 | 1309.881 | 22.679 | 0.000 | 19.191 | 0 |
| precip+elev+hosts | 10 | -2610.689 | -2581.766 | 0.324 | 0.678 | 1309.345 | 23.753 | 0.000 | 18.561 | 0 |
| wind+precip+elev | 10 | -2607.833 | -2578.910 | 0.395 | 0.741 | 1307.917 | 26.609 | 0.000 | 21.127 | 0 |
| temp+precip+hosts | 10 | -2603.941 | -2575.018 | 0.112 | 0.675 | 1305.971 | 30.501 | 0.000 | 21.313 | 0 |
| wind+temp+precip | 10 | -2602.658 | -2573.735 | 0.148 | 0.692 | 1305.329 | 31.784 | 0.000 | 22.612 | 0 |
| temp+elev+hosts | 10 | -2601.746 | -2572.823 | 0.070 | 0.669 | 1304.873 | 32.696 | 0.000 | 22.46 | 0 |
| wind+elev+hosts | 10 | -2601.456 | -2572.533 | 0.081 | 0.682 | 1304.728 | 32.985 | 0.000 | 22.801 | 0 |
| wind+temp+elev | 10 | -2600.857 | -2571.934 | 0.068 | 0.682 | 1304.428 | 33.585 | 0.000 | 24.225 | 0 |
| wind+temp+hosts | 10 | -2600.353 | -2571.430 | 0.050 | 0.675 | 1304.176 | 34.089 | 0.000 | 24.536 | 0 |
| wind+temp+precip+hosts | 13 | -2603.796 | -2549.396 | 0.111 | 0.674 | 1305.898 | 56.122 | 0.000 | 27.761 | 0 |
| temp+precip+elev+hosts | 13 | -2602.961 | -2548.561 | 0.104 | 0.677 | 1305.480 | 56.958 | 0.000 | 28.916 | 0 |
| wind+precip+elev+hosts | 13 | -2602.887 | -2548.487 | 0.118 | 0.688 | 1305.444 | 57.031 | 0.000 | 29.112 | 0 |
| wind+temp+precip+elev | 13 | -2602.879 | -2548.479 | 0.120 | 0.691 | 1305.439 | 57.040 | 0.000 | 29.289 | 0 |
| wind+temp+elev+hosts | 13 | -2600.880 | -2546.480 | 0.059 | 0.678 | 1304.440 | 59.039 | 0.000 | 29.922 | 0 |
| wind+temp+precip+elev+hosts | 16 | -2603.826 | -2502.112 | 0.116 | 0.676 | 1305.913 | 103.407 | 0.000 | 32 | 0 |

| Surface | *k* | *AIC* | *AICc* | *R^2^_m_* | *R^2^_c_* | *LL* | *ΔAICc* | *ω_AICc_* | Rank | B% |
| --- | --- | --- | --- | --- | --- | --- | --- | --- | --- | --- |
| replicate 3 | | | | | | | | | | |
| **precip** | **4** | **-2605.422** | **-2603.317** | **0.448** | **0.757** | **1306.711** | **0.000** | **0.469** | **3.031** | **55.3** |
| **precip+hosts** | **7** | **-2615.340** | **-2602.340** | **0.511** | **0.749** | **1311.670** | **0.977** | **0.288** | **3.466** | **21.9** |
| Distance | 2 | -2596.492 | -2599.921 | 0.027 | 0.684 | 1302.246 | 3.396 | 0.086 | 4.998 | 0.1 |
| hosts | 4 | -2601.359 | -2599.254 | 0.097 | 0.681 | 1304.680 | 4.063 | 0.061 | 4.033 | 20.5 |
| wind | 4 | -2599.833 | -2597.728 | 0.114 | 0.714 | 1303.917 | 5.589 | 0.029 | 5.943 | 2.2 |
| temp | 4 | -2599.711 | -2597.606 | 0.107 | 0.681 | 1303.855 | 5.711 | 0.027 | 5.234 | 0 |
| elev | 4 | -2598.665 | -2596.560 | 0.072 | 0.696 | 1303.333 | 6.757 | 0.016 | 6.77 | 0 |
| NULL | 1 | -2592.124 | -2595.942 | 0.000 | 0.671 | 1299.062 | 7.374 | 0.012 | - | - |
| temp+precip | 7 | -2607.599 | -2594.599 | 0.368 | 0.731 | 1307.799 | 8.718 | 0.006 | 8.56 | 0 |
| wind+precip | 7 | -2606.990 | -2593.990 | 0.469 | 0.763 | 1307.495 | 9.327 | 0.004 | 8.588 | 0 |
| precip+elev | 7 | -2603.031 | -2590.031 | 0.145 | 0.696 | 1305.515 | 13.286 | 0.001 | 11.043 | 0 |
| temp+hosts | 7 | -2602.966 | -2589.966 | 0.069 | 0.672 | 1305.483 | 13.350 | 0.001 | 10.6 | 0 |
| temp+elev | 7 | -2601.652 | -2588.652 | 0.085 | 0.689 | 1304.826 | 14.665 | 0.000 | 12.623 | 0 |
| elev+hosts | 7 | -2601.340 | -2588.340 | 0.096 | 0.681 | 1304.670 | 14.977 | 0.000 | 11.824 | 0 |
| wind+hosts | 7 | -2601.245 | -2588.245 | 0.090 | 0.680 | 1304.623 | 15.072 | 0.000 | 12.413 | 0 |
| wind+elev | 7 | -2600.692 | -2587.692 | 0.086 | 0.701 | 1304.346 | 15.625 | 0.000 | 14.044 | 0 |
| wind+temp | 7 | -2600.651 | -2587.651 | 0.086 | 0.684 | 1304.325 | 15.666 | 0.000 | 13.568 | 0 |
| temp+precip+hosts | 10 | -2615.246 | -2586.322 | 0.481 | 0.732 | 1311.623 | 16.994 | 0.000 | 16.919 | 0 |
| precip+elev+hosts | 10 | -2613.330 | -2584.407 | 0.521 | 0.767 | 1310.665 | 18.910 | 0.000 | 17.792 | 0 |
| wind+precip+hosts | 10 | -2607.823 | -2578.900 | 0.471 | 0.764 | 1307.912 | 24.417 | 0.000 | 20.67 | 0 |
| wind+precip+elev | 10 | -2603.020 | -2574.097 | 0.145 | 0.696 | 1305.510 | 29.220 | 0.000 | 21.413 | 0 |
| temp+precip+elev | 10 | -2602.936 | -2574.013 | 0.144 | 0.697 | 1305.468 | 29.304 | 0.000 | 22.014 | 0 |
| wind+temp+precip | 10 | -2602.667 | -2573.744 | 0.130 | 0.687 | 1305.333 | 29.573 | 0.000 | 21.829 | 0 |
| wind+temp+elev | 10 | -2601.678 | -2572.755 | 0.086 | 0.689 | 1304.839 | 30.562 | 0.000 | 22.747 | 0 |
| temp+elev+hosts | 10 | -2600.964 | -2572.040 | 0.088 | 0.682 | 1304.482 | 31.276 | 0.000 | 23.333 | 0 |
| wind+temp+hosts | 10 | -2600.615 | -2571.692 | 0.061 | 0.676 | 1304.307 | 31.625 | 0.000 | 23.759 | 0 |
| wind+elev+hosts | 10 | -2600.204 | -2571.281 | 0.059 | 0.683 | 1304.102 | 32.036 | 0.000 | 23.786 | 0 |
| wind+precip+elev+hosts | 13 | -2603.538 | -2549.138 | 0.094 | 0.672 | 1305.769 | 54.178 | 0.000 | 27.813 | 0 |
| wind+temp+precip+elev | 13 | -2602.907 | -2548.507 | 0.109 | 0.690 | 1305.454 | 54.809 | 0.000 | 29.014 | 0 |
| temp+precip+elev+hosts | 13 | -2602.694 | -2548.294 | 0.087 | 0.678 | 1305.347 | 55.023 | 0.000 | 28.957 | 0 |
| wind+temp+precip+hosts | 13 | -2602.320 | -2547.920 | 0.135 | 0.690 | 1305.160 | 55.397 | 0.000 | 28.776 | 0 |
| wind+temp+elev+hosts | 13 | -2601.176 | -2546.776 | 0.059 | 0.676 | 1304.588 | 56.541 | 0.000 | 30.44 | 0 |
| wind+temp+precip+elev+hosts | 16 | -2603.814 | -2502.099 | 0.109 | 0.675 | 1305.907 | 101.218 | 0.000 | 32 | 0 |

| Surface | *k* | *AIC* | *AICc* | *R^2^_m_* | *R^2^_c_* | *LL* | *ΔAICc* | *ω_AICc_* | Rank | B% |
| --- | --- | --- | --- | --- | --- | --- | --- | --- | --- | --- |
| replicate 4 | | | | | | | | | | |
| **precip** | **4** | **-2607.228** | **-2605.123** | **0.418** | **0.749** | **1307.614** | **0.000** | **0.731** | **2.741** | **58.4** |
| precip+hosts | 7 | -2613.829 | -2600.829 | 0.435 | 0.714 | 1310.915 | 4.294 | 0.085 | 3.958 | 16.7 |
| Distance | 2 | -2596.492 | -2599.921 | 0.027 | 0.684 | 1302.246 | 5.202 | 0.054 | 5.324 | 0.1 |
| hosts | 4 | -2601.316 | -2599.211 | 0.094 | 0.680 | 1304.658 | 5.912 | 0.038 | 4.287 | 22.5 |
| precip+elev | 7 | -2611.610 | -2598.610 | 0.425 | 0.739 | 1309.805 | 6.513 | 0.028 | 5.718 | 0 |
| temp | 4 | -2600.136 | -2598.030 | 0.086 | 0.677 | 1304.068 | 7.093 | 0.021 | 5.286 | 0.1 |
| wind | 4 | -2599.834 | -2597.728 | 0.113 | 0.713 | 1303.917 | 7.395 | 0.018 | 6.252 | 2.1 |
| elev | 4 | -2598.664 | -2596.558 | 0.069 | 0.694 | 1303.332 | 8.565 | 0.010 | 7.125 | 0.1 |
| NULL | 1 | -2592.124 | -2595.942 | 0.000 | 0.671 | 1299.062 | 9.181 | 0.007 | - | - |
| wind+precip | 7 | -2607.497 | -2594.497 | 0.352 | 0.723 | 1307.748 | 10.626 | 0.004 | 9.085 | 0 |
| temp+precip | 7 | -2605.638 | -2592.638 | 0.274 | 0.697 | 1306.819 | 12.485 | 0.001 | 10.406 | 0 |
| temp+hosts | 7 | -2602.907 | -2589.907 | 0.068 | 0.672 | 1305.454 | 15.216 | 0.000 | 10.836 | 0 |
| temp+elev | 7 | -2601.658 | -2588.658 | 0.071 | 0.680 | 1304.829 | 16.465 | 0.000 | 12.599 | 0 |
| wind+hosts | 7 | -2601.489 | -2588.489 | 0.082 | 0.682 | 1304.745 | 16.634 | 0.000 | 11.954 | 0 |
| wind+elev | 7 | -2600.689 | -2587.689 | 0.088 | 0.702 | 1304.345 | 17.434 | 0.000 | 13.954 | 0 |
| wind+temp | 7 | -2600.603 | -2587.603 | 0.089 | 0.675 | 1304.301 | 17.520 | 0.000 | 13.255 | 0 |
| elev+hosts | 7 | -2600.337 | -2587.337 | 0.094 | 0.686 | 1304.168 | 17.786 | 0.000 | 13.286 | 0 |
| wind+precip+hosts | 10 | -2608.266 | -2579.343 | 0.309 | 0.689 | 1308.133 | 25.780 | 0.000 | 18.351 | 0 |
| temp+precip+hosts | 10 | -2604.277 | -2575.354 | 0.121 | 0.648 | 1306.139 | 29.769 | 0.000 | 19.532 | 0 |
| precip+elev+hosts | 10 | -2603.918 | -2574.995 | 0.110 | 0.675 | 1305.959 | 30.128 | 0.000 | 19.778 | 0 |
| temp+precip+elev | 10 | -2603.203 | -2574.280 | 0.133 | 0.694 | 1305.601 | 30.843 | 0.000 | 21.125 | 0 |
| wind+precip+elev | 10 | -2602.930 | -2574.006 | 0.131 | 0.695 | 1305.465 | 31.117 | 0.000 | 22.212 | 0 |
| wind+temp+precip | 10 | -2602.709 | -2573.786 | 0.109 | 0.688 | 1305.355 | 31.337 | 0.000 | 22.406 | 0 |
| wind+temp+hosts | 10 | -2602.539 | -2573.616 | 0.067 | 0.672 | 1305.270 | 31.507 | 0.000 | 21.622 | 0 |
| temp+elev+hosts | 10 | -2602.100 | -2573.177 | 0.086 | 0.684 | 1305.050 | 31.946 | 0.000 | 23.026 | 0 |
| wind+temp+elev | 10 | -2601.632 | -2572.709 | 0.068 | 0.679 | 1304.816 | 32.414 | 0.000 | 23.74 | 0 |
| wind+elev+hosts | 10 | -2601.428 | -2572.504 | 0.079 | 0.684 | 1304.714 | 32.619 | 0.000 | 23.142 | 0 |
| temp+precip+elev+hosts | 13 | -2603.900 | -2549.500 | 0.114 | 0.676 | 1305.950 | 55.623 | 0.000 | 27.811 | 0 |
| wind+precip+elev+hosts | 13 | -2603.371 | -2548.971 | 0.095 | 0.676 | 1305.686 | 56.152 | 0.000 | 28.745 | 0 |
| wind+temp+precip+hosts | 13 | -2603.124 | -2548.724 | 0.127 | 0.685 | 1305.562 | 56.399 | 0.000 | 28.731 | 0 |
| wind+temp+precip+elev | 13 | -2602.830 | -2548.430 | 0.135 | 0.689 | 1305.415 | 56.693 | 0.000 | 29.144 | 0 |
| wind+temp+elev+hosts | 13 | -2600.311 | -2545.911 | 0.098 | 0.689 | 1304.156 | 59.212 | 0.000 | 30.569 | 0 |
| wind+temp+precip+elev+hosts | 16 | -2602.108 | -2500.394 | 0.115 | 0.686 | 1305.054 | 104.729 | 0.000 | 32 | 0 |

| Surface | *k* | *AIC* | *AICc* | *R^2^_m_* | *R^2^_c_* | *LL* | *ΔAICc* | *ω_AICc_* | Rank | B% |
| --- | --- | --- | --- | --- | --- | --- | --- | --- | --- | --- |
| replicate 5 | | | | | | | | | | |
| **precip** | **4** | **-2606.918** | **-2604.813** | **0.390** | **0.739** | **1307.459** | **0.000** | **0.634** | **2.963** | **56.7** |
| precip+hosts | 7 | -2615.243 | -2602.243 | 0.504 | 0.745 | 1311.622 | 2.569 | 0.176 | 3.588 | 16.1 |
| Distance | 2 | -2596.492 | -2599.921 | 0.027 | 0.684 | 1302.246 | 4.892 | 0.055 | 5.124 | 0 |
| hosts | 4 | -2601.348 | -2599.242 | 0.095 | 0.681 | 1304.674 | 5.570 | 0.039 | 4.125 | 23.6 |
| precip+elev | 7 | -2612.138 | -2599.138 | 0.436 | 0.748 | 1310.069 | 5.674 | 0.037 | 6.351 | 0.1 |
| temp | 4 | -2599.882 | -2597.777 | 0.108 | 0.683 | 1303.941 | 7.036 | 0.019 | 5.279 | 0 |
| wind | 4 | -2599.833 | -2597.728 | 0.114 | 0.714 | 1303.917 | 7.085 | 0.018 | 5.843 | 3 |
| elev | 4 | -2598.664 | -2596.559 | 0.073 | 0.696 | 1303.332 | 8.254 | 0.010 | 6.678 | 0.4 |
| NULL | 1 | -2592.124 | -2595.942 | 0.000 | 0.671 | 1299.062 | 8.870 | 0.008 | - | - |
| wind+precip | 7 | -2606.681 | -2593.681 | 0.209 | 0.686 | 1307.341 | 11.131 | 0.002 | 8.772 | 0.1 |
| temp+precip | 7 | -2602.681 | -2589.681 | 0.159 | 0.694 | 1305.340 | 15.132 | 0.000 | 11.43 | 0 |
| temp+elev | 7 | -2601.756 | -2588.756 | 0.074 | 0.680 | 1304.878 | 16.056 | 0.000 | 12.139 | 0 |
| temp+hosts | 7 | -2601.746 | -2588.746 | 0.071 | 0.671 | 1304.873 | 16.066 | 0.000 | 11.669 | 0 |
| wind+hosts | 7 | -2601.490 | -2588.490 | 0.081 | 0.682 | 1304.745 | 16.323 | 0.000 | 11.525 | 0 |
| wind+elev | 7 | -2600.659 | -2587.659 | 0.088 | 0.703 | 1304.329 | 17.154 | 0.000 | 13.729 | 0 |
| wind+temp | 7 | -2600.653 | -2587.653 | 0.087 | 0.684 | 1304.327 | 17.159 | 0.000 | 13.912 | 0 |
| elev+hosts | 7 | -2600.514 | -2587.514 | 0.075 | 0.680 | 1304.257 | 17.299 | 0.000 | 12.888 | 0 |
| wind+precip+hosts | 10 | -2605.819 | -2576.896 | 0.170 | 0.655 | 1306.910 | 27.916 | 0.000 | 17.943 | 0 |
| precip+elev+hosts | 10 | -2603.849 | -2574.926 | 0.112 | 0.673 | 1305.925 | 29.887 | 0.000 | 19.728 | 0 |
| temp+precip+hosts | 10 | -2603.353 | -2574.429 | 0.095 | 0.673 | 1305.676 | 30.383 | 0.000 | 20.467 | 0 |
| wind+temp+hosts | 10 | -2602.941 | -2574.017 | 0.069 | 0.672 | 1305.470 | 30.795 | 0.000 | 21.05 | 0 |
| temp+precip+elev | 10 | -2602.866 | -2573.943 | 0.164 | 0.700 | 1305.433 | 30.869 | 0.000 | 21.283 | 0 |
| wind+temp+precip | 10 | -2602.806 | -2573.883 | 0.136 | 0.688 | 1305.403 | 30.929 | 0.000 | 21.395 | 0 |
| wind+precip+elev | 10 | -2602.675 | -2573.752 | 0.098 | 0.688 | 1305.337 | 31.061 | 0.000 | 22.501 | 0 |
| temp+elev+hosts | 10 | -2602.073 | -2573.150 | 0.072 | 0.682 | 1305.037 | 31.663 | 0.000 | 22.955 | 0 |
| wind+elev+hosts | 10 | -2601.434 | -2572.511 | 0.078 | 0.684 | 1304.717 | 32.302 | 0.000 | 22.817 | 0 |
| wind+temp+elev | 10 | -2600.779 | -2571.856 | 0.073 | 0.692 | 1304.389 | 32.957 | 0.000 | 24.846 | 0 |
| wind+temp+precip+hosts | 13 | -2603.913 | -2549.513 | 0.111 | 0.675 | 1305.956 | 55.300 | 0.000 | 27.732 | 0 |
| temp+precip+elev+hosts | 13 | -2603.310 | -2548.910 | 0.127 | 0.688 | 1305.655 | 55.902 | 0.000 | 28.57 | 0 |
| wind+precip+elev+hosts | 13 | -2602.938 | -2548.538 | 0.141 | 0.690 | 1305.469 | 56.274 | 0.000 | 28.735 | 0 |
| wind+temp+precip+elev | 13 | -2602.652 | -2548.252 | 0.118 | 0.685 | 1305.326 | 56.560 | 0.000 | 29.467 | 0 |
| wind+temp+elev+hosts | 13 | -2599.446 | -2545.046 | 0.046 | 0.681 | 1303.723 | 59.767 | 0.000 | 30.496 | 0 |
| wind+temp+precip+elev+hosts | 16 | -2603.010 | -2501.296 | 0.086 | 0.678 | 1305.505 | 103.516 | 0.000 | 32 | 0 |

| Surface | *k* | *AIC* | *AICc* | *R^2^_m_* | *R^2^_c_* | *LL* | *ΔAICc* | *ω_AICc_* | Rank | B% |
| --- | --- | --- | --- | --- | --- | --- | --- | --- | --- | --- |
| replicate 6 | | | | | | | | | | |
| **precip** | **4** | **-2608.282** | **-2606.177** | **0.406** | **0.741** | **1308.141** | **0.000** | **0.773** | **2.631** | **59** |
| precip+hosts | 7 | -2615.574 | -2602.574 | 0.507 | 0.744 | 1311.787 | 3.603 | 0.128 | 3.446 | 15.7 |
| Distance | 2 | -2596.492 | -2599.921 | 0.027 | 0.684 | 1302.246 | 6.256 | 0.034 | 5.095 | 0.1 |
| hosts | 4 | -2601.359 | -2599.253 | 0.099 | 0.681 | 1304.679 | 6.924 | 0.024 | 4.218 | 22.1 |
| temp | 4 | -2600.147 | -2598.041 | 0.094 | 0.680 | 1304.073 | 8.136 | 0.013 | 5.033 | 0.5 |
| wind | 4 | -2599.833 | -2597.728 | 0.112 | 0.713 | 1303.917 | 8.449 | 0.011 | 5.991 | 2 |
| elev | 4 | -2598.663 | -2596.558 | 0.073 | 0.696 | 1303.332 | 9.619 | 0.006 | 6.836 | 0.6 |
| NULL | 1 | -2592.124 | -2595.942 | 0.000 | 0.671 | 1299.062 | 10.235 | 0.005 | - | - |
| precip+elev | 7 | -2607.965 | -2594.965 | 0.438 | 0.754 | 1307.983 | 11.212 | 0.003 | 8.057 | 0 |
| wind+precip | 7 | -2607.422 | -2594.422 | 0.358 | 0.720 | 1307.711 | 11.755 | 0.002 | 8.305 | 0 |
| temp+precip | 7 | -2603.323 | -2590.323 | 0.257 | 0.707 | 1305.662 | 15.854 | 0.000 | 11.285 | 0 |
| temp+hosts | 7 | -2602.902 | -2589.902 | 0.066 | 0.671 | 1305.451 | 16.275 | 0.000 | 10.964 | 0 |
| temp+elev | 7 | -2601.695 | -2588.695 | 0.088 | 0.689 | 1304.848 | 17.482 | 0.000 | 12.537 | 0 |
| wind+hosts | 7 | -2601.488 | -2588.488 | 0.081 | 0.682 | 1304.744 | 17.689 | 0.000 | 12.078 | 0 |
| elev+hosts | 7 | -2600.901 | -2587.901 | 0.083 | 0.678 | 1304.451 | 18.276 | 0.000 | 12.611 | 0 |
| wind+elev | 7 | -2600.687 | -2587.687 | 0.086 | 0.701 | 1304.343 | 18.490 | 0.000 | 13.814 | 0 |
| wind+temp | 7 | -2600.543 | -2587.543 | 0.091 | 0.677 | 1304.272 | 18.634 | 0.000 | 13.187 | 0 |
| temp+precip+hosts | 10 | -2610.787 | -2581.864 | 0.386 | 0.707 | 1309.394 | 24.313 | 0.000 | 17.668 | 0 |
| wind+precip+hosts | 10 | -2607.094 | -2578.171 | 0.337 | 0.712 | 1307.547 | 28.006 | 0.000 | 19.904 | 0 |
| precip+elev+hosts | 10 | -2603.759 | -2574.836 | 0.134 | 0.687 | 1305.880 | 31.341 | 0.000 | 20.17 | 0 |
| temp+precip+elev | 10 | -2603.285 | -2574.362 | 0.128 | 0.689 | 1305.643 | 31.815 | 0.000 | 21.141 | 0 |
| wind+precip+elev | 10 | -2602.886 | -2573.963 | 0.133 | 0.692 | 1305.443 | 32.214 | 0.000 | 22.145 | 0 |
| temp+elev+hosts | 10 | -2602.660 | -2573.737 | 0.066 | 0.671 | 1305.330 | 32.440 | 0.000 | 21.392 | 0 |
| wind+temp+precip | 10 | -2602.574 | -2573.651 | 0.148 | 0.692 | 1305.287 | 32.526 | 0.000 | 22.156 | 0 |
| wind+elev+hosts | 10 | -2601.463 | -2572.540 | 0.080 | 0.683 | 1304.731 | 33.637 | 0.000 | 22.667 | 0 |
| wind+temp+elev | 10 | -2601.166 | -2572.243 | 0.071 | 0.685 | 1304.583 | 33.934 | 0.000 | 23.769 | 0 |
| wind+temp+hosts | 10 | -2600.774 | -2571.851 | 0.062 | 0.678 | 1304.387 | 34.326 | 0.000 | 23.9 | 0 |
| wind+temp+precip+hosts | 13 | -2603.035 | -2548.635 | 0.087 | 0.673 | 1305.517 | 57.542 | 0.000 | 28.296 | 0 |
| wind+temp+precip+elev | 13 | -2602.963 | -2548.563 | 0.131 | 0.692 | 1305.481 | 57.614 | 0.000 | 28.47 | 0 |
| temp+precip+elev+hosts | 13 | -2602.252 | -2547.852 | 0.146 | 0.686 | 1305.126 | 58.325 | 0.000 | 28.661 | 0 |
| wind+precip+elev+hosts | 13 | -2601.027 | -2546.627 | 0.076 | 0.667 | 1304.514 | 59.550 | 0.000 | 29.289 | 0 |
| wind+temp+elev+hosts | 13 | -2599.756 | -2545.356 | 0.054 | 0.688 | 1303.878 | 60.821 | 0.000 | 30.284 | 0 |
| wind+temp+precip+elev+hosts | 16 | -2602.364 | -2500.650 | 0.077 | 0.681 | 1305.182 | 105.527 | 0.000 | 32 | 0 |

| Surface | *k* | *AIC* | *AICc* | *R^2^_m_* | *R^2^_c_* | *LL* | *ΔAICc* | *ω_AICc_* | Rank | B% |
| --- | --- | --- | --- | --- | --- | --- | --- | --- | --- | --- |
| replicate 7 | | | | | | | | | | |
| **precip** | **4** | **-2605.531** | **-2603.425** | **0.425** | **0.753** | **1306.765** | **0.000** | **0.609** | **3.103** | **65.6** |
| Distance | 2 | -2596.492 | -2599.921 | 0.027 | 0.684 | 1302.246 | 3.504 | 0.106 | 5.182 | 0.2 |
| hosts | 4 | -2601.394 | -2599.288 | 0.097 | 0.681 | 1304.697 | 4.137 | 0.077 | 3.944 | 23.6 |
| precip+elev | 7 | -2611.539 | -2598.539 | 0.427 | 0.744 | 1309.770 | 4.886 | 0.053 | 5.689 | 1.2 |
| temp | 4 | -2600.109 | -2598.003 | 0.097 | 0.682 | 1304.054 | 5.422 | 0.041 | 4.921 | 0.1 |
| wind+precip | 7 | -2610.823 | -2597.823 | 0.414 | 0.740 | 1309.412 | 5.602 | 0.037 | 6.512 | 4.5 |
| wind | 4 | -2599.828 | -2597.723 | 0.117 | 0.716 | 1303.914 | 5.703 | 0.035 | 5.923 | 3.2 |
| elev | 4 | -2598.624 | -2596.519 | 0.095 | 0.706 | 1303.312 | 6.907 | 0.019 | 6.934 | 0.4 |
| NULL | 1 | -2592.124 | -2595.942 | 0.000 | 0.671 | 1299.062 | 7.483 | 0.014 | - | - |
| temp+precip | 7 | -2606.600 | -2593.600 | 0.338 | 0.725 | 1307.300 | 9.825 | 0.004 | 9.698 | 0 |
| precip+hosts | 7 | -2604.547 | -2591.547 | 0.158 | 0.661 | 1306.273 | 11.878 | 0.002 | 9.063 | 1.2 |
| temp+hosts | 7 | -2602.911 | -2589.911 | 0.070 | 0.672 | 1305.455 | 13.515 | 0.001 | 10.837 | 0 |
| temp+elev | 7 | -2601.760 | -2588.760 | 0.074 | 0.679 | 1304.880 | 14.665 | 0.000 | 12.364 | 0 |
| wind+hosts | 7 | -2601.490 | -2588.490 | 0.082 | 0.682 | 1304.745 | 14.936 | 0.000 | 12.334 | 0 |
| elev+hosts | 7 | -2601.115 | -2588.115 | 0.086 | 0.680 | 1304.558 | 15.310 | 0.000 | 12.691 | 0 |
| wind+elev | 7 | -2600.674 | -2587.674 | 0.086 | 0.701 | 1304.337 | 15.751 | 0.000 | 14.158 | 0 |
| wind+temp | 7 | -2600.646 | -2587.646 | 0.085 | 0.684 | 1304.323 | 15.780 | 0.000 | 13.744 | 0 |
| temp+precip+hosts | 10 | -2615.488 | -2586.564 | 0.515 | 0.750 | 1311.744 | 16.861 | 0.000 | 17.364 | 0 |
| precip+elev+hosts | 10 | -2615.122 | -2586.199 | 0.484 | 0.735 | 1311.561 | 17.227 | 0.000 | 17.685 | 0 |
| wind+precip+hosts | 10 | -2612.254 | -2583.331 | 0.406 | 0.707 | 1310.127 | 20.095 | 0.000 | 18.757 | 0 |
| temp+precip+elev | 10 | -2604.779 | -2575.856 | 0.217 | 0.685 | 1306.390 | 27.569 | 0.000 | 21.392 | 0 |
| wind+temp+precip | 10 | -2602.881 | -2573.958 | 0.135 | 0.691 | 1305.441 | 29.467 | 0.000 | 22.432 | 0 |
| wind+precip+elev | 10 | -2602.731 | -2573.808 | 0.144 | 0.695 | 1305.365 | 29.618 | 0.000 | 22.638 | 0 |
| temp+elev+hosts | 10 | -2601.835 | -2572.912 | 0.070 | 0.679 | 1304.917 | 30.514 | 0.000 | 23.392 | 0 |
| wind+temp+hosts | 10 | -2601.830 | -2572.907 | 0.067 | 0.670 | 1304.915 | 30.519 | 0.000 | 23.018 | 0 |
| wind+temp+elev | 10 | -2601.642 | -2572.719 | 0.072 | 0.682 | 1304.821 | 30.706 | 0.000 | 23.956 | 0 |
| wind+elev+hosts | 10 | -2601.432 | -2572.509 | 0.079 | 0.684 | 1304.716 | 30.916 | 0.000 | 23.269 | 0 |
| temp+precip+elev+hosts | 13 | -2604.995 | -2550.595 | 0.123 | 0.656 | 1306.497 | 52.830 | 0.000 | 27.674 | 0 |
| wind+precip+elev+hosts | 13 | -2603.634 | -2549.234 | 0.131 | 0.687 | 1305.817 | 54.192 | 0.000 | 28.325 | 0 |
| wind+temp+precip+elev | 13 | -2602.893 | -2548.493 | 0.134 | 0.693 | 1305.446 | 54.933 | 0.000 | 29.372 | 0 |
| wind+temp+precip+hosts | 13 | -2602.773 | -2548.373 | 0.134 | 0.686 | 1305.386 | 55.052 | 0.000 | 29.161 | 0 |
| wind+temp+elev+hosts | 13 | -2600.013 | -2545.613 | 0.062 | 0.678 | 1304.007 | 57.812 | 0.000 | 30.468 | 0 |
| wind+temp+precip+elev+hosts | 16 | -2603.167 | -2501.453 | 0.121 | 0.685 | 1305.583 | 101.973 | 0.000 | 32 | 0 |

| Surface | *k* | *AIC* | *AICc* | *R^2^_m_* | *R^2^_c_* | *LL* | *ΔAICc* | *ω_AICc_* | Rank | B% |
| --- | --- | --- | --- | --- | --- | --- | --- | --- | --- | --- |
| replicate 8 | | | | | | | | | | |
| **precip** | **4** | **-2604.979** | **-2602.873** | **0.443** | **0.755** | **1306.489** | **0.000** | **0.468** | **3.438** | **54.9** |
| precip+hosts | 7 | -2613.404 | -2600.404 | 0.465 | 0.733 | 1310.702 | 2.469 | 0.136 | 4.192 | 19.5 |
| Distance | 2 | -2596.492 | -2599.921 | 0.027 | 0.684 | 1302.246 | 2.953 | 0.107 | 5.496 | 0.1 |
| hosts | 4 | -2601.145 | -2599.040 | 0.099 | 0.681 | 1304.573 | 3.834 | 0.069 | 4.458 | 21.5 |
| precip+elev | 7 | -2611.931 | -2598.931 | 0.395 | 0.730 | 1309.966 | 3.942 | 0.065 | 5.989 | 1.6 |
| temp | 4 | -2600.168 | -2598.063 | 0.089 | 0.679 | 1304.084 | 4.811 | 0.042 | 5.453 | 0.2 |
| wind+precip | 7 | -2610.996 | -2597.996 | 0.421 | 0.741 | 1309.498 | 4.878 | 0.041 | 6.975 | 0.1 |
| wind | 4 | -2599.822 | -2597.716 | 0.107 | 0.711 | 1303.911 | 5.157 | 0.035 | 6.347 | 1.8 |
| elev | 4 | -2598.648 | -2596.542 | 0.085 | 0.701 | 1303.324 | 6.331 | 0.020 | 7.247 | 0.3 |
| NULL | 1 | -2592.124 | -2595.942 | 0.000 | 0.671 | 1299.062 | 6.931 | 0.015 | - | - |
| temp+hosts | 7 | -2602.967 | -2589.967 | 0.069 | 0.672 | 1305.483 | 12.907 | 0.001 | 10.477 | 0 |
| temp+precip | 7 | -2602.636 | -2589.636 | 0.131 | 0.687 | 1305.318 | 13.237 | 0.001 | 11.372 | 0 |
| temp+elev | 7 | -2601.675 | -2588.675 | 0.068 | 0.679 | 1304.837 | 14.199 | 0.000 | 12.535 | 0 |
| wind+hosts | 7 | -2601.491 | -2588.491 | 0.081 | 0.683 | 1304.745 | 14.383 | 0.000 | 12.302 | 0 |
| elev+hosts | 7 | -2601.336 | -2588.336 | 0.096 | 0.681 | 1304.668 | 14.537 | 0.000 | 12.032 | 0 |
| wind+elev | 7 | -2600.691 | -2587.691 | 0.088 | 0.703 | 1304.346 | 15.182 | 0.000 | 14.156 | 0 |
| wind+temp | 7 | -2600.645 | -2587.645 | 0.087 | 0.684 | 1304.323 | 15.228 | 0.000 | 13.746 | 0 |
| temp+precip+hosts | 10 | -2613.490 | -2584.567 | 0.480 | 0.742 | 1310.745 | 18.307 | 0.000 | 17.694 | 0 |
| wind+precip+hosts | 10 | -2606.542 | -2577.619 | 0.141 | 0.651 | 1307.271 | 25.254 | 0.000 | 19.608 | 0 |
| wind+precip+elev | 10 | -2606.473 | -2577.550 | 0.433 | 0.755 | 1307.236 | 25.324 | 0.000 | 20.399 | 0 |
| precip+elev+hosts | 10 | -2606.311 | -2577.388 | 0.234 | 0.676 | 1307.155 | 25.486 | 0.000 | 20.35 | 0 |
| temp+precip+elev | 10 | -2604.802 | -2575.879 | 0.381 | 0.741 | 1306.401 | 26.994 | 0.000 | 21.599 | 0 |
| wind+temp+precip | 10 | -2602.667 | -2573.744 | 0.133 | 0.688 | 1305.333 | 29.130 | 0.000 | 22.196 | 0 |
| wind+temp+hosts | 10 | -2601.958 | -2573.035 | 0.059 | 0.671 | 1304.979 | 29.839 | 0.000 | 22.42 | 0 |
| temp+elev+hosts | 10 | -2601.788 | -2572.865 | 0.069 | 0.676 | 1304.894 | 30.009 | 0.000 | 22.998 | 0 |
| wind+temp+elev | 10 | -2601.721 | -2572.798 | 0.074 | 0.679 | 1304.860 | 30.076 | 0.000 | 23.342 | 0 |
| wind+elev+hosts | 10 | -2600.887 | -2571.964 | 0.079 | 0.690 | 1304.444 | 30.909 | 0.000 | 24.179 | 0 |
| wind+precip+elev+hosts | 13 | -2603.836 | -2549.436 | 0.105 | 0.674 | 1305.918 | 53.438 | 0.000 | 28.007 | 0 |
| temp+precip+elev+hosts | 13 | -2603.139 | -2548.739 | 0.103 | 0.660 | 1305.570 | 54.134 | 0.000 | 28.116 | 0 |
| wind+temp+precip+elev | 13 | -2602.847 | -2548.447 | 0.122 | 0.687 | 1305.424 | 54.426 | 0.000 | 29.226 | 0 |
| wind+temp+precip+hosts | 13 | -2602.555 | -2548.155 | 0.090 | 0.678 | 1305.277 | 54.719 | 0.000 | 29.988 | 0 |
| wind+temp+elev+hosts | 13 | -2602.330 | -2547.930 | 0.069 | 0.674 | 1305.165 | 54.944 | 0.000 | 29.663 | 0 |
| wind+temp+precip+elev+hosts | 16 | -2600.949 | -2499.234 | 0.053 | 0.673 | 1304.474 | 103.639 | 0.000 | 32 | 0 |

| Surface | *k* | *AIC* | *AICc* | *R^2^_m_* | *R^2^_c_* | *LL* | *ΔAICc* | *ω_AICc_* | Rank | B% |
| --- | --- | --- | --- | --- | --- | --- | --- | --- | --- | --- |
| replicate 9 | | | | | | | | | | |
| **precip+hosts** | **7** | **-2615.723** | **-2602.723** | **0.496** | **0.737** | **1311.862** | **0.000** | **0.443** | **3.183** | **29.2** |
| **precip** | **4** | **-2603.530** | **-2601.425** | **0.424** | **0.748** | **1305.765** | **1.298** | **0.232** | **3.583** | **45.5** |
| Distance | 2 | -2596.492 | -2599.921 | 0.027 | 0.684 | 1302.246 | 2.802 | 0.109 | 5.325 | 0.2 |
| hosts | 4 | -2601.380 | -2599.275 | 0.096 | 0.681 | 1304.690 | 3.449 | 0.079 | 4.114 | 21.8 |
| wind | 4 | -2599.831 | -2597.726 | 0.116 | 0.715 | 1303.915 | 4.998 | 0.036 | 6.064 | 2.9 |
| temp | 4 | -2599.571 | -2597.466 | 0.104 | 0.692 | 1303.785 | 5.258 | 0.032 | 5.727 | 0.1 |
| elev | 4 | -2598.665 | -2596.559 | 0.071 | 0.695 | 1303.332 | 6.164 | 0.020 | 7.098 | 0.3 |
| precip+elev | 7 | -2609.196 | -2596.196 | 0.400 | 0.736 | 1308.598 | 6.527 | 0.017 | 7.366 | 0 |
| NULL | 1 | -2592.124 | -2595.942 | 0.000 | 0.671 | 1299.062 | 6.781 | 0.015 | - | - |
| temp+precip | 7 | -2608.214 | -2595.214 | 0.402 | 0.739 | 1308.107 | 7.509 | 0.010 | 8.294 | 0 |
| wind+precip | 7 | -2605.976 | -2592.976 | 0.374 | 0.740 | 1306.988 | 9.747 | 0.003 | 10.115 | 0 |
| temp+hosts | 7 | -2602.909 | -2589.909 | 0.066 | 0.671 | 1305.454 | 12.814 | 0.001 | 10.83 | 0 |
| temp+elev | 7 | -2601.615 | -2588.615 | 0.064 | 0.675 | 1304.807 | 14.108 | 0.000 | 12.526 | 0 |
| wind+hosts | 7 | -2601.437 | -2588.437 | 0.080 | 0.683 | 1304.719 | 14.286 | 0.000 | 12.45 | 0 |
| elev+hosts | 7 | -2601.232 | -2588.232 | 0.095 | 0.681 | 1304.616 | 14.491 | 0.000 | 12.092 | 0 |
| wind+elev | 7 | -2600.688 | -2587.688 | 0.088 | 0.703 | 1304.344 | 15.035 | 0.000 | 13.95 | 0 |
| wind+temp | 7 | -2600.651 | -2587.651 | 0.085 | 0.675 | 1304.326 | 15.072 | 0.000 | 13.343 | 0 |
| wind+precip+hosts | 10 | -2604.589 | -2575.666 | 0.108 | 0.664 | 1306.294 | 27.058 | 0.000 | 18.881 | 0 |
| temp+precip+hosts | 10 | -2604.512 | -2575.589 | 0.166 | 0.666 | 1306.256 | 27.135 | 0.000 | 18.933 | 0 |
| precip+elev+hosts | 10 | -2603.585 | -2574.662 | 0.094 | 0.673 | 1305.792 | 28.062 | 0.000 | 19.862 | 0 |
| temp+precip+elev | 10 | -2603.583 | -2574.659 | 0.104 | 0.667 | 1305.791 | 28.064 | 0.000 | 20.614 | 0 |
| wind+precip+elev | 10 | -2603.129 | -2574.206 | 0.128 | 0.691 | 1305.564 | 28.518 | 0.000 | 21.117 | 0 |
| wind+temp+precip | 10 | -2602.487 | -2573.563 | 0.101 | 0.682 | 1305.243 | 29.160 | 0.000 | 21.979 | 0 |
| wind+temp+elev | 10 | -2601.729 | -2572.806 | 0.074 | 0.679 | 1304.864 | 29.917 | 0.000 | 22.926 | 0 |
| wind+temp+hosts | 10 | -2601.364 | -2572.441 | 0.065 | 0.671 | 1304.682 | 30.283 | 0.000 | 22.93 | 0 |
| temp+elev+hosts | 10 | -2601.135 | -2572.212 | 0.078 | 0.694 | 1304.567 | 30.511 | 0.000 | 24.225 | 0 |
| wind+elev+hosts | 10 | -2600.974 | -2572.051 | 0.081 | 0.693 | 1304.487 | 30.673 | 0.000 | 23.473 | 0 |
| wind+precip+elev+hosts | 13 | -2604.118 | -2549.718 | 0.116 | 0.681 | 1306.059 | 53.005 | 0.000 | 27.657 | 0 |
| wind+temp+precip+elev | 13 | -2603.133 | -2548.733 | 0.117 | 0.691 | 1305.566 | 53.990 | 0.000 | 29.013 | 0 |
| temp+precip+elev+hosts | 13 | -2602.576 | -2548.176 | 0.153 | 0.697 | 1305.288 | 54.547 | 0.000 | 28.945 | 0 |
| wind+temp+precip+hosts | 13 | -2602.527 | -2548.127 | 0.070 | 0.671 | 1305.263 | 54.596 | 0.000 | 28.995 | 0 |
| wind+temp+elev+hosts | 13 | -2600.741 | -2546.341 | 0.069 | 0.678 | 1304.371 | 56.382 | 0.000 | 30.39 | 0 |
| wind+temp+precip+elev+hosts | 16 | -2603.575 | -2501.861 | 0.094 | 0.674 | 1305.788 | 100.862 | 0.000 | 32 | 0 |

| Surface | *k* | *AIC* | *AICc* | *R^2^_m_* | *R^2^_c_* | *LL* | *ΔAICc* | *ω_AICc_* | Rank | B% |
| --- | --- | --- | --- | --- | --- | --- | --- | --- | --- | --- |
| replicate 10 | | | | | | | | | | |
| **precip+hosts** | **7** | **-2615.984** | **-2602.984** | **0.527** | **0.754** | **1311.992** | **0.000** | **0.513** | **2.899** | **69.2** |
| precip | 4 | -2602.446 | -2600.341 | 0.156 | 0.694 | 1305.223 | 2.643 | 0.137 | 3.663 | 5.3 |
| Distance | 2 | -2596.492 | -2599.921 | 0.027 | 0.684 | 1302.246 | 3.063 | 0.111 | 5.421 | 0.2 |
| hosts | 4 | -2601.294 | -2599.189 | 0.092 | 0.680 | 1304.647 | 3.795 | 0.077 | 4.334 | 21.3 |
| temp | 4 | -2600.041 | -2597.936 | 0.100 | 0.681 | 1304.020 | 5.048 | 0.041 | 5.564 | 0.3 |
| wind | 4 | -2599.834 | -2597.728 | 0.113 | 0.714 | 1303.917 | 5.255 | 0.037 | 6.435 | 2.9 |
| precip+elev | 7 | -2610.408 | -2597.408 | 0.453 | 0.753 | 1309.204 | 5.576 | 0.032 | 6.445 | 0.3 |
| elev | 4 | -2598.665 | -2596.560 | 0.072 | 0.696 | 1303.332 | 6.424 | 0.021 | 7.328 | 0.4 |
| NULL | 1 | -2592.124 | -2595.942 | 0.000 | 0.671 | 1299.062 | 7.041 | 0.015 | - | - |
| wind+precip | 7 | -2608.750 | -2595.750 | 0.458 | 0.760 | 1308.375 | 7.234 | 0.014 | 7.752 | 0.1 |
| temp+precip | 7 | -2603.105 | -2590.105 | 0.413 | 0.746 | 1305.553 | 12.878 | 0.001 | 10.924 | 0 |
| temp+hosts | 7 | -2602.931 | -2589.931 | 0.069 | 0.672 | 1305.466 | 13.052 | 0.001 | 10.866 | 0 |
| temp+elev | 7 | -2601.700 | -2588.700 | 0.086 | 0.688 | 1304.850 | 14.283 | 0.000 | 12.618 | 0 |
| elev+hosts | 7 | -2601.308 | -2588.308 | 0.094 | 0.681 | 1304.654 | 14.675 | 0.000 | 12.246 | 0 |
| wind+hosts | 7 | -2601.283 | -2588.283 | 0.095 | 0.681 | 1304.642 | 14.700 | 0.000 | 12.383 | 0 |
| wind+elev | 7 | -2600.682 | -2587.682 | 0.087 | 0.702 | 1304.341 | 15.302 | 0.000 | 14 | 0 |
| wind+temp | 7 | -2600.651 | -2587.651 | 0.087 | 0.684 | 1304.325 | 15.333 | 0.000 | 13.458 | 0 |
| precip+elev+hosts | 10 | -2614.656 | -2585.733 | 0.440 | 0.723 | 1311.328 | 17.251 | 0.000 | 17.568 | 0 |
| wind+precip+elev | 10 | -2608.912 | -2579.989 | 0.281 | 0.693 | 1308.456 | 22.995 | 0.000 | 19.367 | 0 |
| temp+precip+hosts | 10 | -2604.470 | -2575.547 | 0.128 | 0.659 | 1306.235 | 27.437 | 0.000 | 20.231 | 0 |
| wind+precip+hosts | 10 | -2603.919 | -2574.995 | 0.109 | 0.675 | 1305.959 | 27.988 | 0.000 | 20.491 | 0 |
| temp+elev+hosts | 10 | -2602.994 | -2574.071 | 0.067 | 0.673 | 1305.497 | 28.913 | 0.000 | 21.516 | 0 |
| temp+precip+elev | 10 | -2602.824 | -2573.900 | 0.110 | 0.686 | 1305.412 | 29.083 | 0.000 | 22.264 | 0 |
| wind+temp+precip | 10 | -2602.659 | -2573.736 | 0.108 | 0.688 | 1305.329 | 29.248 | 0.000 | 22.794 | 0 |
| wind+temp+hosts | 10 | -2602.074 | -2573.151 | 0.073 | 0.674 | 1305.037 | 29.833 | 0.000 | 22.042 | 0 |
| wind+elev+hosts | 10 | -2601.147 | -2572.224 | 0.076 | 0.689 | 1304.574 | 30.759 | 0.000 | 23.736 | 0 |
| wind+temp+elev | 10 | -2600.746 | -2571.823 | 0.074 | 0.694 | 1304.373 | 31.161 | 0.000 | 24.655 | 0 |
| wind+temp+precip+hosts | 13 | -2603.538 | -2549.138 | 0.098 | 0.675 | 1305.769 | 53.846 | 0.000 | 27.976 | 0 |
| wind+precip+elev+hosts | 13 | -2603.331 | -2548.931 | 0.094 | 0.678 | 1305.666 | 54.052 | 0.000 | 28.771 | 0 |
| wind+temp+precip+elev | 13 | -2603.006 | -2548.606 | 0.124 | 0.688 | 1305.503 | 54.377 | 0.000 | 28.875 | 0 |
| temp+precip+elev+hosts | 13 | -2602.779 | -2548.379 | 0.113 | 0.695 | 1305.390 | 54.604 | 0.000 | 29.652 | 0 |
| wind+temp+elev+hosts | 13 | -2601.913 | -2547.513 | 0.063 | 0.672 | 1304.956 | 55.471 | 0.000 | 29.726 | 0 |
| wind+temp+precip+elev+hosts | 16 | -2602.437 | -2500.723 | 0.083 | 0.684 | 1305.218 | 102.261 | 0.000 | 32 | 0 |
